# Delineation of the heterogeneity underlying genomic instability in hereditary breast cancers reveals four disease subtypes

**DOI:** 10.1038/s12276-026-01693-4

**Published:** 2026-04-16

**Authors:** Sunmin Kim, Seeyoun Lee, Hyeji Kim, Su Jung Kang, Heejung Chae, Bong-Jo Kim, Jinhwa Kong, Min-Chae Kang, Tae-Min Kim, Sun-Young Kong

**Affiliations:** 1 Department of Medical Informatics, The Catholic University of Korea College of Medicine, Seoul, Republic of Korea; 2 Department of Surgery, Center for Breast Cancer, National Cancer Center, Goyang, Republic of Korea; 3 Targeted Therapy Branch, National Cancer Center, Goyang, Republic of Korea; 4 Department of Cancer Biomedical Science, National Cancer Center Graduate School of Cancer Science and Policy, Goyang, Republic of Korea; 5 Department of Biomedicine & Health Sciences, Graduate School, The Catholic University of Korea, Seoul, Republic of Korea; 6 Department of Medical Oncology, Center for Breast Cancer, National Cancer Center, Goyang, Republic of Korea; 7 Department of Internal Medicine, Seoul National University Bundang Hospital, Seoul National University, College of Medicine, Seongnam, Republic of Korea; 8 Division of Genome Science, Department of Precision Medicine, National Institute of Health, Cheongju, Republic of Korea; 9 Division of Healthcare and Artificial Intelligence, Department of Precision Medicine, National Institute of Health, Cheongju, Republic of Korea; 10 Cancer Research Institute, College of Medicine, The Catholic University of Korea, Seoul, Republic of Korea; 11 CMC Institute for Basic Medical Science, The Catholic Medical Center of the Catholic University of Korea, Seoul, Republic of Korea; 12 Department of Laboratory Medicine, National Cancer Center, Goyang, Republic of Korea

**Keywords:** Breast cancer, Cancer genomics

## Abstract

Hereditary breast cancers (hBCs) exhibit genomic heterogeneity, but their underlying genomic instability and clinical implications remain unclear. We conducted whole-genome sequencing on 129 *BRCA1/2*-negative hBCs, with subsets analyzed by methylome and transcriptome sequencing, and integrated COSMIC mutational, copy number (CN), and structural variation signatures. We identified four subtypes: homologous recombination-deficient (HRD), mutation-dominant (MUT), CN-dominant, and genome stable. HRD tumors exhibited genomic instability, including promoter hypermethylation and loss of heterozygosity at *BRCA1/2*. Some HRD-low hBC genomes showed elevated mutations and CN changes, indicating HRD-independent mechanisms. MUT tumors showed high tumor mutation burdens with APOBEC-associated kataegis and cytolytic/immune-activation programs with M1 macrophage including upregulation of GZMA, GZMB, and large-scale transition. CN tumors were enriched for small-scale loss of heterozygosity maintaining euploidy with recurrent focal losses implicating classic tumor suppressors. Transcriptomics indicated immune and stromal infiltration in MUT and CN subtypes, respectively, suggesting subtype-specific therapeutic vulnerabilities. Functional analysis in cell lines suggests poly (ADP-ribose) polymerase inhibitors and cytotoxic chemotherapy sensitivity in HRD and CN tumors, whereas immune features in MUT tumors support vulnerability to immunotherapy. These findings suggest that distinct hBC subtypes delineated by genomic instability can advance insights into molecular heterogeneity beyond expression-based classifications and support an integrative genomic instability index (HRD score, ploidy, size-stratified CN burden, and signature exposures) for patient stratification and personalized therapeutic strategies.

## Distinct subtypes of hereditary breast cancer revealed

Breast cancer is a common cancer in women, and some cases are hereditary. This study looks at patients with breast cancer without BRCA1/BRCA2 mutations to find other genetic causes. Researchers used whole-genome sequencing on 129 patients to explore this. They found that although many patients showed genomic instability, it was not always due to known BRCA mutations. They identified four subtypes of breast cancer based on different patterns of DNA changes: homologous recombination-deficient, mutation-dominant, copy number, and genome stable. The study used advanced techniques such as whole-genome sequencing to identify these patterns. This approach helps understand how different genetic changes can lead to breast cancer. The findings suggest that different subtypes may respond differently to treatments, such as poly (ADP-ribose) polymerase inhibitors or chemotherapy. This research could lead to more personalized treatment options for patients with breast cancer in the future.

This summary was initially drafted using artificial intelligence, then revised and fact-checked by the author.

## Introduction

Breast cancer is one of the most prevalent malignancies and the primary cause of cancer-related deaths in women^1,2^. Approximately 5–10% of invasive cases are hereditary breast cancers (hBCs)^3^. Women with a first-degree relative with breast cancer have a twofold increased risk of developing the disease^4^. Pathogenic germline variants in the well-known breast cancer-related genes, *BRCA1*/*BRCA2*, account for 15–25% of hBC cases^5^ but other highly penetrant and pathogenic genetic variants have been identified. These include genes involved in DNA damage sensing (for example, *ATM*, *ATR*, *CHEK1*, and *CHEK2*) and those in the homologous recombination repair (HRR) pathway (for example, *MRE11*, *RAD50*, *NSB1*, *BARD1*, *BLM*, *BRIP1*, *PALB2*, and *RAD51B*/*C*/*D*). Collectively, germline variants in these genes account for ~50% of hBC cases not explained by *BRCA1* or *BRCA2* mutations (*BRCA1/BRCA2*-negative hBC)^6^. Genes associated with inherited cancer syndromes, such as *TP53*, *PTEN*, *STK11*, *NF1*, and *CDH1*, are also implicated in increased risk of hBCs^7^. Despite the heterogeneity of genes involved in the development of hBCs, their functional losses collectively result in homologous recombination deficiency (HRD), leading to the genomic instability and susceptibility to chemotherapy and poly (ADP-ribose) polymerase inhibitor (PARPi)^8,9^. The genomic consequences of HRD, known as chromosomal scars, can be quantified through HRD scores, which have been proposed to predict the effectiveness of PARPi in breast^10^ and ovarian cancers^11^. However, further investigations are needed to clarify the heterogeneity of HRD and to define the extent and types of genomic instability driven by HRD in *BRCA1/BRCA2*-negative hBCs.

Mutations in cancer genomes often exhibit unique sequence properties that reflect underlying mutational processes^12,13^. Through mutational signature analysis, these processes can be identified within individual cancer genomes. For example, single-base substitution (SBS) mutation signature 3 (SBS3) is associated with HRD, and genomes exhibiting HRD show higher SBS3 abundance than genomes without HRD^14,15^. Previous studies have shown that combining SBS3 abundance with HRD scores provides a more accurate prediction of HRD in cancer genomes^16^. Originally developed to analyze single-nucleotide variants (SNVs) or point mutations based on SBS signatures, the scope of mutation signatures has expanded to include double-base substitutions, insertions and deletions (indels), and large-scale alterations such as somatic copy number (CN) alterations and structural variations (SVs). Thus, whole-genome sequencing (WGS) that can identify all these types of alterations can offer valuable insights into types of genomic instability and the activity of specific DNA repair in individual cancer genomes. However, it remains unclear whether detection of additional genomic instability beyond HRD in breast cancers can clarify the heterogeneity of potential genomic instability mechanisms in hBC genomes. Dissecting the types and extents of genomic instability that are operative in hBC genomes can also facilitate the molecular classification of hBCs, potentially defining new subtypes based on distinct genomic instability mechanisms beyond the current transcription-based molecular subtypes^17^.

In this study, we performed WGS on 129 patients with hBC lacking germline *BRCA1/BRCA2* pathogenic variants to identify pathogenic variants beyond *BRCA* mutations and to dissect the heterogeneity of genomic instability. Our analysis revealed that although HRD is a major driver of genomic instability, other mechanisms also contribute to overall genomic instability in hBC genomes. Our analysis led to the identification of four distinct hBC subtypes based on prevalent genomic alterations: HRD, mutation-dominant (MUT), and CN subtypes (each dominated by high HRD scores, mutations, and CNs, respectively), along with genome stable (GS) subtypes. This refined classification enhances our understanding of hBC subtypes by integrating genomic instability and mutation signature analysis. In vitro pharmacological data from cell lines suggest that this heterogeneity may correspond to varying responses to treatments, with HRD subtypes showing greater susceptibility to PARPi, and CN subtypes responding better to platinum or taxane-based chemotherapy.

## Materials and methods

### Study population

This retrospective analysis included 129 female patients with breast cancer from a National Cancer Center in Korea (IRB No. NCC2021-0144). All of them underwent germline clinical genetic testing between May 2013 and July 2021 owing to suspected genetic predisposition. The indications for clinical germline genetic testing were defined by the Korean insurance reimbursement criteria for BRCA1/2 testing, which included: (i) breast cancer diagnosed before age 40; (ii) breast cancer with a family history of breast or ovarian cancer among second-degree relatives; (iii) a personal history of both breast and other cancers; (iv) bilateral breast cancer; and (iv) male breast cancer. For this study, we defined hBC as patients with breast cancer fulfilling established clinical genetic testing criteria for hereditary cancer, regardless of a presence of pathogenic germline variants. The classifications of molecular subtypes were based on the 2011 St Gallen Consensus^18^.

### Whole-genome sequencing

DNA was extracted using a QIAamp DNA Blood Mini Kit (Qiagen, Hilden, Germany). Library was prepared using the TruSeq DNA PCR-Free Library Prep Kit (Illumina, San Diego, CA, USA), quality-checked on a Bioanalyzer 2100 (Agilent Technologies, Santa Clara, CA, USA), and quantified on the QX200 Droplet Digital PCR System (Bio-Rad, Hercules, CA, USA). Indexed libraries were pooled equimolarly and sequenced on an Illumina NovaSeq 6000 system, following the manufacturer’s protocol for paired-end 150 bp reads (2 × 150 bp). Details on the sequencing information are available in Supplementary Table 1.

### Data processing and mutation analyses

The FASTQ files were processed following the Genome Analysis Toolkit (GATK) Best Practices guidelines (https://gatk.broadinstitute.org/)^19^. Sequencing reads were initially aligned to the hg19 reference genome using BWA-MEM (v0.7.17)^20^. Subsequently, local realignment and base quality score recalibration were performed using GATK (v4.1.7.0). Sequencing read processing, including duplicate removal and sorting, was carried out using SAMtools (v1.13)^21^ and Picard (v2.27.1, http://broadinstitute.github.io/picard/). Mutations, including SNVs and short indels, were identified using MuTect2 (v2.2) in GATK by comparing tumor and matched normal sequencing data^19^. The identified SNVs and indels were annotated with ANNOVAR (v2019Oct24)^22^. Overall, comparison of tumor and matched normal genomes (with average sequencing depths of 106.4948 and 34.15695, respectively) identified 728,595 SNVs and 112,126 indels using MuTect2. Among 129 patients, the number of SNVs ranged from 281 to 38,219 (median 3,263) and that of indels ranged from 247 to 3,399 (median 640). A list of coding SNVs and indels is provided in Supplementary Table 2. Kataegis, defined as localized hypermutations with at least six consecutive SNVs less than 100 bp apart, was also assessed per case^23^. Kataegis-related mutations are further classified based on six substitution features (C > A, C > G, C > T, T > A, T > C, and T > G) for single-nucleotide substitutions and 10 substitution features (AC > NN, AT > NN, CC > NN, CG > NN, CT > NN, GC > NN, TA > NN, TC > NN, TG > NN, and TT > NN) for di-nucleotide substitutions. Kataegis mutation clusters were identified and visualized using the karyoploteR package based on the criterion of short inter-mutation distances (less than 2 kb apart) between mutations^23^.

### Germline variants

Germline variants were identified using HaplotypeCaller (v4.1.7.0) in GATK using WGS data from matched normal or blood DNA samples^19^. The interpretation and classification of these variants followed the ACMG/AMP guidelines^24^. On the basis of the five-tier classification system of pathogenicity, we included variants classified as pathogenic and likely pathogenic. Variants of uncertain significance (VUS) were further evaluated for their impact on the encoded amino acids using ANNOVAR (v2019Oct24)^22^. Nonsense variants, splice site variants, and frameshift indels were considered likely pathogenic and included in the list. For VUS, we considered as ‘damaging’ by SIFT (Sorting Intolerant From Tolerant)^25^ and PolyPhen2 (ref. ^26^). The filtered set of germline variants is available in Supplementary Table 3.

### CN alterations and SVs

Somatic CN alterations were identified using ascatNGS (v4.5.0) to detect allele-specific CN changes from WGS data^27^. Log ratios and B-allele frequencies of polymorphic markers estimated by ascatNGS were subject to smoothing using the Circular Binary Segmentation algorithm (v1.72.3)^28^. Tumor purity and ploidy for each case were also derived from the ascatNGS output. CN segments were classified into categories, including loss of heterozygosity (LOH), homozygous deletions, and heterozygous CNs, along with the assignment of integer-level CNs by ascatNGS. Integer-level CN profiles along with tumor purity and ploidy were analyzed using scarHRD^29^ to derive three measures of HRD: number of telomeric allelic imbalance^30^, large-scale transition (LST), and LOH. HRD scores were defined as the unweighted sum of three measures. Whole-genome duplication was called for cases with a ploidy level greater than 3.5 as estimated by ascatNGS. Recurrent CNs were identified using GISTIC analysis (v2.0.23)^31^. CN profiles with genomic coordinates are available in Supplementary Table 4.

SVs were detected using DELLY software (v1.3.3) and classified into deletions, duplications, inversions, and chromosomal translocations^32^. SVs were further subclassified as clustered or non-clustered and also by size to derive features for SV signature analysis. Chromothripsis events were identified using the ShatterSeek R package (v1.1)^33^, following established criteria for classification as high-confidence or low-confidence events^34^. Annotated SVs are available in Supplementary Table 5.

### Mutation signature analysis and signature-based taxonomy

Mutational features required for mutation signature analyses, that is, SBS, double-base substitutions, indels, CN variations, and SV, were identified using SigProfilerExtractor (v1.2.1)^35^ based on the feature properties provided by the COSMICv3.1 database (https://cancer.sanger.ac.uk/signatures/). Details on the abundance or exposure of the five types of mutation signatures are available in Supplementary Table 6.

### mRNA sequencing and transcriptome analysis

RNA was extracted from tissue. Purity was assessed a NanoDrop 2000 spectrophotometer (Thermo Fisher Scientific, Waltham, MA, USA) and integrity by RNA Integrity Number using a Bioanalyzer 2100 (Agilent Technologies, Santa Clara, CA, USA). mRNA libraries were prepared with the TruSeq Stranded mRNA Library Prep kit (Illumina), verified by TapeStation (Agilent Technologies), quantified by quantitative PCR (qPCR) using KAPA SYBR FAST qPCR Master Mix (Kapa Biosystems, Wilmington, MA, USA), pooled equimolarly, and sequenced on Illumina NovaSeq 6000 system following the provided protocols for 2 × 100 bp sequencing. Raw RNA-seq reads were aligned using STAR aligner (v2.7.11a)^36^, and gene-level read counts were prepared by HTseq (v0.13.5)^37^. Gene-level expression profiles were normalized into transcripts per million (TPM) for subsequent analyses and are available in Supplementary Table 7.

To identify differentially expressed genes among the subtypes, the edgeR package (v4.7.3) was used to estimate both statistical significance (false discovery rate) and expression fold changes^38^. For pathway-level analysis, we performed gene set enrichment analysis using the fgsea R package (v2.12) with 1000 permutations^39^. The Reactome gene set from the Molecular Signatures Database (MsigDB) served as the reference for functional annotation^40^, and pathways with *P*-values < 0.05 were regarded as significantly enriched. Immune infiltration scores were computed using the ESTIMATE algorithm (v1.10.13)^41^ to assess the immune landscape of each transcriptome data. For deconvolution of bulk-level RNA-sequencing, we used CIBERSORTx, a computational method to analyze cellular composition based on available cell-type-specific signatures^42^. The deconvolution was performed using the TR4 signature matrix representing immune (CD45^+^), stromal (CD10^+^ and CD31^+^), and epithelial (EPCAM) cells to estimate the relative abundance of four cell types. In addition, an LM22 signature matrix, representing 22 immune cell subtypes, was used to impute the relative abundance of 22 immune cells. The cell-type-specific expression profiles were imputed using a non-negative least-squares regression algorithm and the inferred cellular abundance^42^. The cell-type-specific expression of four cell types corresponding to those of TR4 was inferred across four hBC subtypes. A functional enrichment analysis for cell-type-specific expression was performed using a gene set variant analysis (GSVA) algorithm^43^. GSVA was conducted using the R package GSVA (v1.34) and hallmark gene sets from the MSigDB^39^.

### Human methylome sequencing

Genomic DNA was enriched using the Twist Human Methylome panel (Twist Bioscience, San Francisco, CA, USA). Libraries were prepared using a NEBNext Enzymatic Methyl-seq Methylation Library Preparation Kit (New England Biolabs, MA, USA), amplified using Q5U (a modified version of Q5 High-Fidelity DNA Polymerase), and quality-checked on a TapeStation (Agilent Technologies). After qPCR using KAPA SYBR FAST qPCR Master Mix (Kapa Biosystems, Wilmington, MA, USA), we pooled index-tagged libraries in equimolar amounts. Sequencing was performed using an Illumina NovaSeq 6000 system (Illumina), following the provided protocols for 2 × 100 sequencing.

Reads were aligned onto hg19 reference genomes using the methylation-aware aligner of Bismark^44^ (v0.22.3), generating methylation calls for each cytosine. Coverage values were extracted alongside the methylation levels for the specified regions. Beta values (M/cov) were calculated for each sample, distinguishing between tumor and normal samples, specifically in gene promoter regions defined as 1000 bp upstream and 400 bp downstream of the transcription start site. Differential methylation analysis across samples was conducted using the methylSig R package^45^.

### Drug sensitivity

To evaluate subtype-specific therapeutic implication, drug sensitivity data were obtained from two independent sources. The PRISM Repurposing data set of Cancer Cell Line Encyclopedia (CCLE), assessed via DepMap (Broad Institute), provided effect values expressed as log2 of fold change for nine drugs. These included five PARPi (olaparib, talazoparib, veliparib, niraparib, and rucaparib), three platinum-based (cisplatin, carboplatin, and oxaliplatin), and one taxane-based (paclitaxel) drug. These drugs were evaluated across 51 breast cancer cell lines. The classification of hBC subtypes for CCLE lines was performed using the same genomic criteria based on HRD scores, tumor mutational burden (TMB), and CN burden. Detailed genomic features and subtype annotations for each CCLE breast cancer cell line are provided in Supplementary Table 8.

Experimental validation was performed using IC50-based drug assays on 11 breast cancer cell lines. Breast cancer cells with *BRCA1*/*2* mutant (BT-474, MDA-MB-436, HCC-1937, HCC-1569, and HCC-1395) and cells with BRCA1/2 wild-type (MDA-MB-231, MDA-MB-453, HCC-70, MCF-7, SK-BR-3, and T47D) were treated with olaparib (Chemscene, Monmouth Junction, NJ, USA), talazoparib (Selleckchem, Houston, TX, USA), cisplatin (Chemscene), paclitaxel (Chemscene), docetaxel (Chemscene), and carboplatin (Chemscene) at various concentrations. Cell viability was measured using the CellTiter-Glo 2.0 (Promega, Madison, WI, USA) assay after 72 h of drug exposure, and dose–response curves were fitted to determine IC50 values.

## Results

### Genomic heterogeneity in BRCA1/2-negative hereditary breast cancers reveals diverse patterns of homologous recombination deficiency and instability

**Fig. 1:**
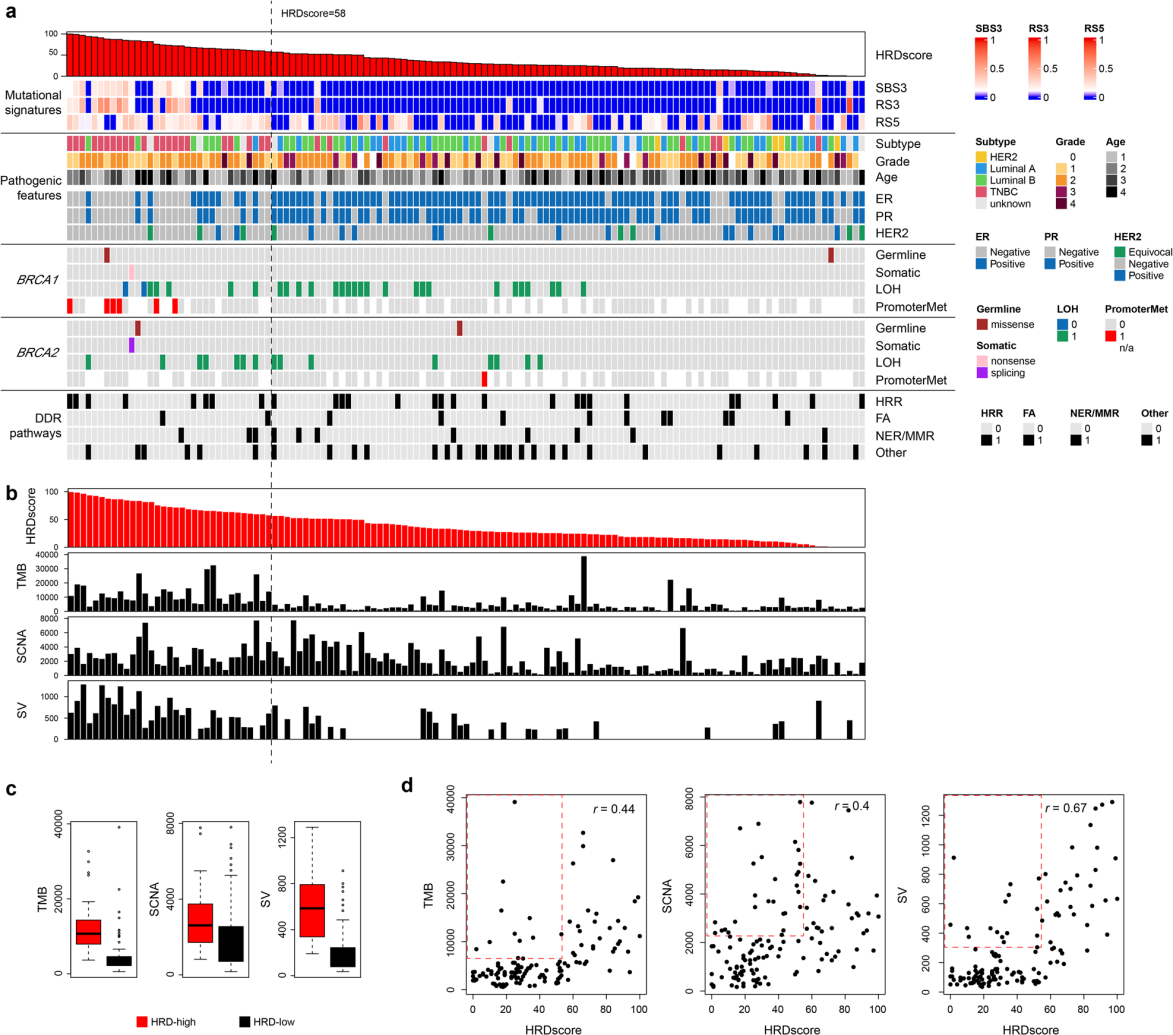
Genomic and clinical characteristics associated with BRCAness and HRD score. **a** Samples are sorted by homologous recombination deficiency (HRD) score. The top panel shows the levels of HRD-associated mutational signatures (single-base substitution 3 (SBS3), RS3, and RS5), which are highly enriched in samples with HRD score ≥58. Below, clinical and pathological features including clinical subtype, grade, and age are shown. Notably, triple-negative breast cancer (TNBC) is more prevalent in the HRD-high group. Consistent with TNBC characteristics, a large proportion of samples in this group is negative for estrogen receptor (ER), progesterone receptor (PR), and HER2 expression. Further down, alterations in *BRCA1* and *BRCA2*, including germline mutations, somatic mutations, loss of heterozygosity (LOH), and promoter methylation, are displayed. BRCA1 tumor/normal differentially methylated region with tumor methylation >0.2 is frequently observed in HRD-high samples. The bottom panel presents detected germline and somatic variants in DNA damage repair (DDR) pathway genes. **b** Bar plots show HRD score, tumor mutational burden (TMB), somatic copy number alteration (SCNA) count, and structural variant (SV) count. All three genomic features are associated with higher HRD scores. **c** Comparing HRD-high and HRD-low groups reveals significantly higher levels of TMB, SCNA, and SVs in the HRD-high group (*t*-test *P* = 4.15 × 10^−7^, 0.004, and 5.74 × 10^−9^, respectively). **d** Scatter plots of TMB, SCNA, and SV counts versus HRD score demonstrate positive correlations (Pearson’s *r* = 0.44, 0.40, and 0.67, respectively). However, a subset of samples with low HRD scores also displays high TMB, SCNA, or SV levels (highlighted in red boxes). RS, RNA single base substitution, HER Human Epidermal Growth Factor Receptor, HRR homologous recombination repair, FA Fanconi anemia, NER Nucleotide Excision Repair, MMR Mismatch Repair.

A total of 129 patients with hBC negative for inherited pathogenic *BRCA1/ BRCA2* variants were enrolled (Fig. 1a, see Materials and methods), and their clincopathological features are summarized in Supplementary Table 9. WGS was performed on tumor-normal pairs at mean depth of 100× and 60×, respectively (Supplementary Table 1). Using WGS data, we obtained four types of somatic alterations of SNV, short indels, CN alterations, and SV. Somatic alterations in hBC genomes are available in Supplementary Tables 2, 4, and 5. For 80 patients with sufficient tissue, whole-genome bisulfite sequencing and transcriptome sequencing were also conducted (Supplementary Table 7). Germline variants were called from the matched normal genomic DNA with pathogenic germline variants listed in Supplementary Table 3.

Figure 1a illustrates the major genomic features of 129 hBC genomes relative to the HRD level. HRD score was calculated as an unweighted sum of three chromosomal indices: LST, telomeric allelic imbalance, and LOH (HRD-LOH)^29^. As previously reported in hBC^46,47^, HRD scores correlated with genomic and clinicopathological features (Fig. 1a). For example, mutation signatures known to be associated with HRD, SBS3, RS3, and RS5 SBS and rearrangement signatures previously used for breast cancers^12^ showed strong correlation with HRD scores. The abundance of HRD-related mutation signatures (SBS3 and RS5) largely discriminated 33 HRD-high and 96 HRD-low hBC genomes using a HRD score cut-off of 58, which was determined through integrated analyses of clinical and genomic features including TMB, somatic CN alterations, and SVs. Clinically, HRD-high genomes are enriched for triple-negative breast cancers (TNBCs) and high-grade tumors, in line with prior findings^48,49^.

In addition to one *BRCA1* (p.Glu1609Ter) and one *BRCA2* somatic truncating variant (p.Ala2603LeufsTer45), frequent LOH events occurred for *BRCA1*/*BRCA2*. Of note, promoter hypermethylation of *BRCA1* is exclusively observed for high-HRD hBC genomes. To identify candidate germline variants associated with hBC beyond *BRCA1*/*BRCA2*, we examined the genes involved in the HRR pathway or those with DNA damage-sensing roles that harbored potentially damaging germline variants. We observed potentially damaging germline variants in *BLM* (*n* = 7), *BARD1* (*n* = 7), *TP53BP1* (*n* = 3), *ATR* (*n* = 3), *ATM* (*n* = 3), *RAD51D* (*n* = 3), *BRCA1* (*n* = 2), and *BRCA2* (*n* = 2). These variants, previously implicated in hBC familial risks, were observed in 22.5% of the patients. At the time of initial germline clinical genetic testing, four patients with germline VUS in *BRCA1* (*n* = 2) and *BRCA2* (*n* = 2), as well as other patients, were confirmed negative for pathogenic BRCA1 or BRCA2 variants. The variants were classified according to the American College of Medical Genetics (ACMG)/Association for Molecular Pathology (AMP) guidelines and cross-referenced with the ClinVar database. Following a ClinVar database update (ver20240715), one of the *BRCA2* variants has been reclassified as pathogenic variant/likely pathogenic variant (PV/LPV), whereas the remaining three variants are classified as VUS. According to the ACMG/AMP guidelines, the classifications of the four variants are: one likely pathogenic and three VUS. Additional DNA damage repair (DDR)-related genes with frequent potentially damaging germline variants included *FANCL* (*n* = 4), *FANCM* (*n* = 2), *FANCI* (*n* = 2), *FANCD2* (*n* = 2, all in Fanconi anemia pathway), *POLE* (*n* = 4), *ERCC2* (*n* = 3, both in the nucleotide excision repair pathway), and *MLH3* (*n* = 3, in the mismatch repair pathway). Furthermore, potentially damaging variants on other DDR pathways were common in *ASCC3* (*n* = 7), *PPP4R2* (*n* = 6), *NEIL3* (*n* = 6), *RAD9A* (*n* = 6), *SMARCC1* (*n* = 5), and *PARP4* (*n* = 5), suggesting contribution to hBC risk. Thus, hBC genomes negative for pathogenic *BRCA1/2* germline variants may produce germline defects in other HRR-related or DNA repair-related genes.

We next investigated the relationships between HRD scores and three genomic instability measures: number of somatic mutations (that is, TMB), CN segments, and SV breakpoints (Fig. 1b). All three measures were significantly higher in HRD-high genomes compared with HRD-low genomes (*P* = 4.15 × 10^−7^, 2.94 × 10^−6^, and 7.02 × 10^−8^ for TMB, CN, and SV, respectively, *t*-test; Fig. 1c). This suggests that HRD contributes to an overall increase in these genomic alterations. However, a subset of HRD-low genomes also exhibited elevated TMB, along with increased numbers of CN segments and SV breakpoints (Fig. 1b,c). This heterogeneity is further emphasized in scatter plots showing the relationships between HRD score and genomic instability measures (Fig. 1d). Despite an overall trend, several hBC genomes displayed high burdens of TMB, CN, and SV without a corresponding increase in HRD score (open rectangles in Fig. 1d). Overall, these findings underscore the underlying heterogeneity in the mechanisms driving genomic instability in breast cancer genomes, independent of HRD status.

### Genomic-instability-based hBC taxonomy identifies distinct subtypes

**Fig. 2:**
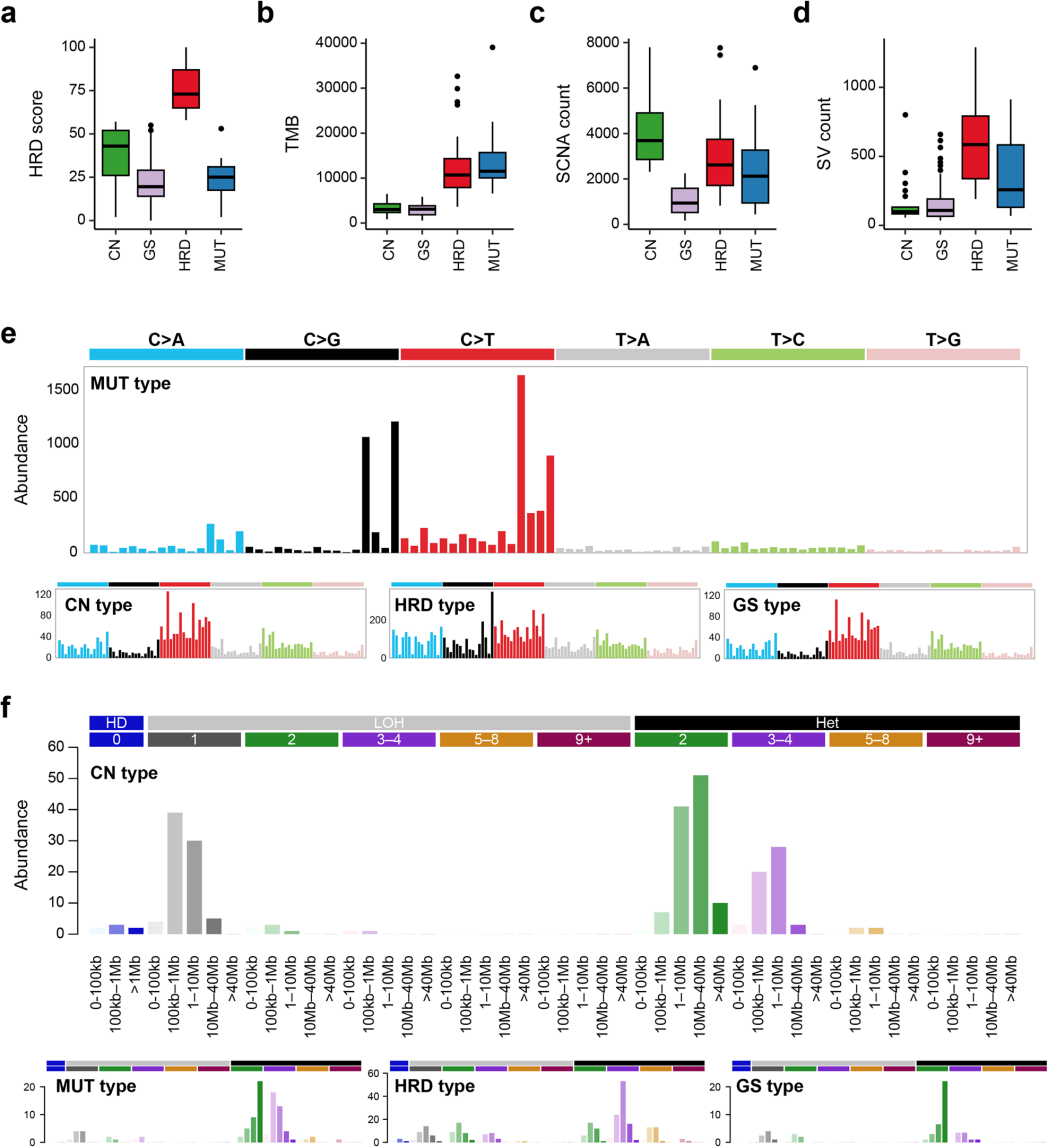
Genomic alteration patterns and mutational signatures across four hereditary breast cancer subtypes. Boxplots comparing genomic features across four molecular subtypes: homologous recombination deficient (HRD), mutation-dominant (MUT), copy number (CN)-dominant, and genome stable (GS). **a** HRD score is significantly highest in the HRD subtype. **b** Tumor mutational burden (TMB) is highest in the MUT subtype. **c** Somatic copy number alterations (SCNAs) are most frequent in the CN subtype. **d** Structural variant (SV) count is highest in the HRD subtype. **a–d** Across all panels, the GS subtype consistently shows the lowest levels of all genomic alterations. **e** Single-base substitution (SBS) mutational signature profiles specific to MUT subtype. Unlike the other three subtypes, which exhibit similar SBS patterns with varying magnitudes, the MUT subtype displays a distinct SBS signature composition. In particular, trinucleotide substitutions such as T[C > G]A, T[C > G]T, T[C > T]A, and T[C > T]T were significantly enriched, representing the well-established genomic footprints of APOBEC deaminase activity. **f** Signature profile of the CN subtype reveals an enrichment of focal loss of heterozygosity (LOH) events, particularly those with CN = 1 and segment lengths between 100 kb and 10 Mb. HD Homozygous deletions, HET heterozygous.

To cope with the heterogeneity underlying genomic instability beyond HRD, we stratified the genomes into four distinct subtypes based on the abundance of major types of genomic alterations, that is, HRD scores, TMB, and the burdens of CN and SV. Genomes with elevated HRD scores and concurrent increases in TMB, CN alterations, and SVs were classified as the ‘HRD’ subtype (*n* = 33; Fig. 2a). Among the genomes with low HRD scores, those exhibiting TMB above the cohort mean (TMB > 2.2 mut/Mb) were categorized as the ‘MUT’ subtype (*n* = 11; Fig. 2b), whereas those characterized by CN above the cohort mean (seg > 2,269) were designated the ‘CN’ subtype (*n* = 25; Fig. 2c). The remaining genomes exhibiting consistently low levels of the measured genomic instability were defined as the GS subtype (Fig. 2a–d). Among the types of alterations, the abundance of SV exhibited a strong correlation with HRD score (*r* = 0.44, 0.40, and 0.67 for TMB, CN, and SV, respectively). Elevated SVs were predominantly observed in the HRD subtype across all cases. Thus, four hBC subtypes were defined under the assumption that genomic instability in hBC genomes can emerge independent of HRD, indicating that distinct and heterogeneous mutational processes were operative in hBC genomes.

Comparison with known molecular subtypes of breast cancers^17^ revealed that only the HRD subtype consistently corresponds to TNBC and the basal-like subtype (Supplementary Fig. 1). This suggests that previous driver-based or gene expression-based molecular classification of breast cancers may not capture the heterogeneity underlying the genomic instability in hBC genomes. To further characterize the distinct mutational features, we analyzed the mutation signatures of hBC subtypes. In the MUT subtype, specific trinucleotide base substitutions, including T[C > G]A, T[C > G]T, T[C > T]A, and T[C > T]T, were significantly enriched. These patterns are well-established genomic footprints of APOBEC deaminase activity (Fig. 2e). In the CN subtype, CN-related mutational signatures indicated a high frequency of LOH events smaller than 10 Mb, particularly in the 100 kb–10 Mb ranges. These included numerous single-copy losses and, to a lesser extent, homozygous deletions (Fig. 2f). The hBC subtype-specific mutation signature-related features are presented in Supplementary Fig. 2. These findings suggest that, in *BRCA1/2*-negative hBC subtypes, diverse mechanisms drive genomic instability that are not fully captured by expression-based classifications; this four-class taxonomy refines hBC molecular heterogeneity and indicates the need for subtype-specific therapeutic strategies.

### MUT subtype of hBC genomes is characterized by APOBEC mutagenesis and kataegis

**Fig. 3:**
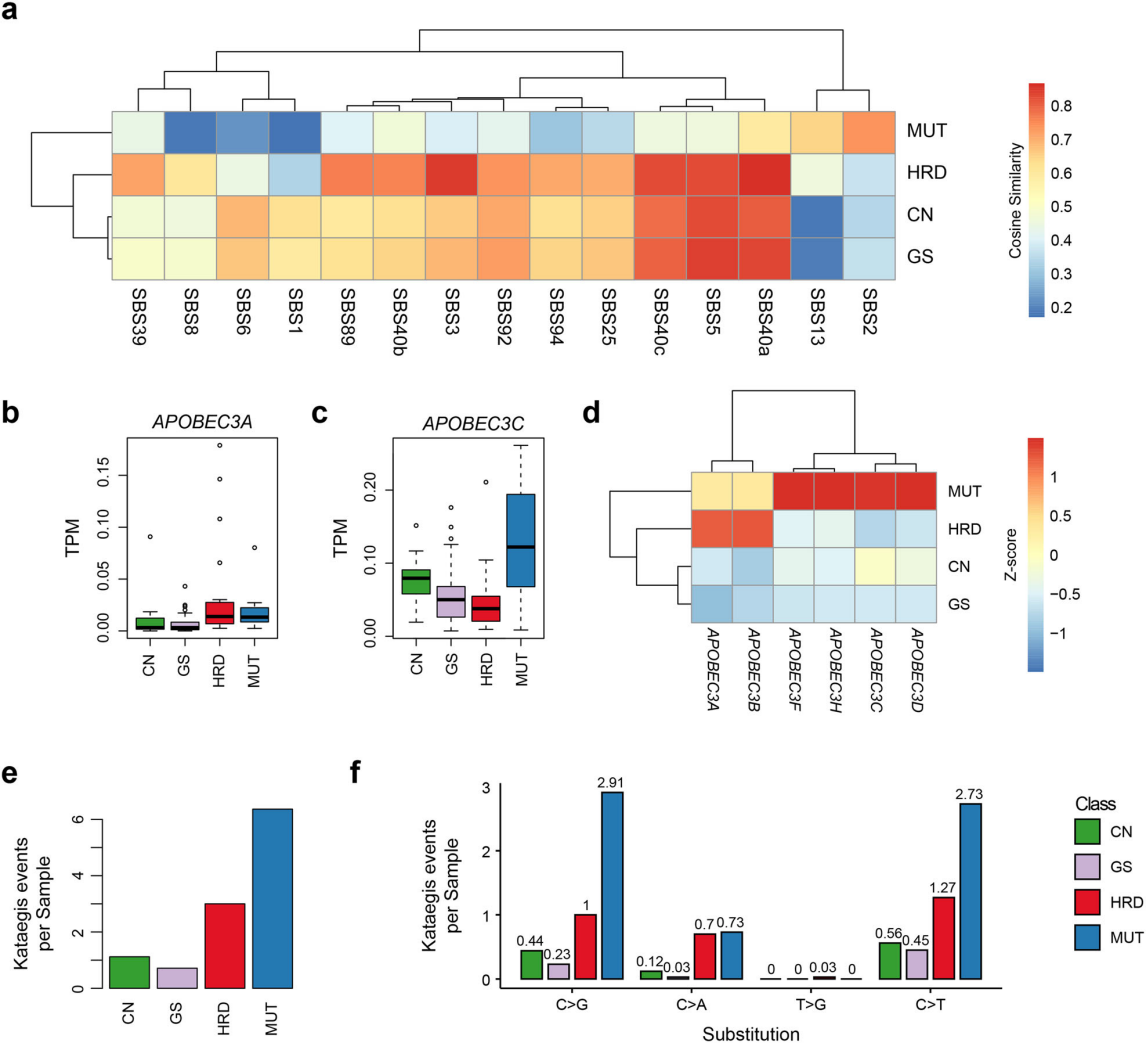
APOBEC-associated mutational features in the MUT subtype. **a** Cosine similarity analysis between each patient’s single-base substitution (SBS) profile and 86 COSMIC SBS signatures. Only signatures with similarity >0.6 in at least one subtype are shown. The mutation-dominant (MUT) subtype exhibits the highest similarity with SBS2 (0.736) and SBS13 (0.648), which are APOBEC-associated signatures. By contrast, the homologous recombination deficient (HRD) subtype shows greater similarity with HRD-associated signatures, SBS3 (0.848) and SBS40a (0.867). **b** Expression levels (transcripts per million (TPM)) of *APOBEC3A* across subtypes. Although the HRD subtype shows the highest median TPM, overall expression levels are low and comparable between HRD and MUT subtypes. **c** Expression of *APOBEC3C* across subtypes reveals a heightened presence of the MUT subtype compared with other subtypes, although the difference was not significant. **d** *Z*-score normalized expression of APOBEC family genes (*APOBEC3A*, *3B*, *3C*, *3D*, *3F*, and *3H*) by subtype. The MUT subtype shows elevated expression across the APOBEC3 family, with *APOBEC3A* and *3B* being higher in the HRD subtype. **e** Bar plot showing the number of kataegis events per sample, with the highest frequency observed in the MUT subtype. **f** Distribution of kataegis events by substitution type. C > G and C > T substitutions are the most frequently observed, consistent with APOBEC-related hypermutation patterns. CN copy number, GS genome stable.

To investigate the mutational features of hBC subtypes relative to known mutation signatures, we estimated the relative abundance of COSMIC SBS signatures using deconstructSigs^50^. Figure 3a shows the distribution of all 86 COSMIC SBS signatures associated with at least one group (>0.6 cosine similarity). Consistent with Fig. 2e, the majority of SNVs in the MUT subtype hBC genomes is attributed to SBS2 and SBS13. These signatures preferentially target TpCpW trinucleotides, leading to C > T and C > G substitutions as features linked to APOBEC deaminase activity^51^. SNVs attributed to SBS2 and SBS13 comprise a median of 27.0% (range 12.5–37.0%) of mutations in MUT subtype hBC genomes, compared with 0% (median, range 0–1.5%) in non-MUT subtype. In addition, we observed that HRD subtypes are enriched for SBS3, a signature previously associated with BRCA deficiency, along with the time-clock signatures SBS5 and SBS40 (ref. ^12^). Notably, MUT subtype tumors exhibit markedly low exposure to SBS5/SBS40. Coupled with their strong SBS2/SBS13, this indicates that their mutational landscape is driven primarily by APOBEC activity rather than age-associated background mutagenesis.

We next assessed the expression of the APOBEC family genes. Although the canonical *APOBEC3A*, frequently observed in cancers, did not show significant transcriptional upregulation across hBC subtypes (Fig. 3b), other APOBEC genes, such as *APOBEC3C*, exhibited transcriptional upregulation in the MUT subtype compared with the HRD subtype (Fig. 3c,d). Although both *APOBEC3A/APOBEC3B* are major mutational drivers in breast cancer genomes^52^, our data suggest that APOBEC activity may be subtype-specific and may involve non-canonical APOBEC enzymes, warranting further investigation. APOBEC-induced mutations often occur in localized hypermutated clusters, known as kataegis, typically spanning ~10 kb (ref. ^52^). In our data set, kataegis events were most frequent in the MUT subtype hBC genomes (Fig. 3e,f), and the events were characterized by clusters of C > G and C > T substitutions consistent with known APOBEC activity (Fig. 3f and Supplementary Fig. 3).

### CN and HRD subtypes of hBC genomes exhibit distinct chromosomal instability

**Fig. 4:**
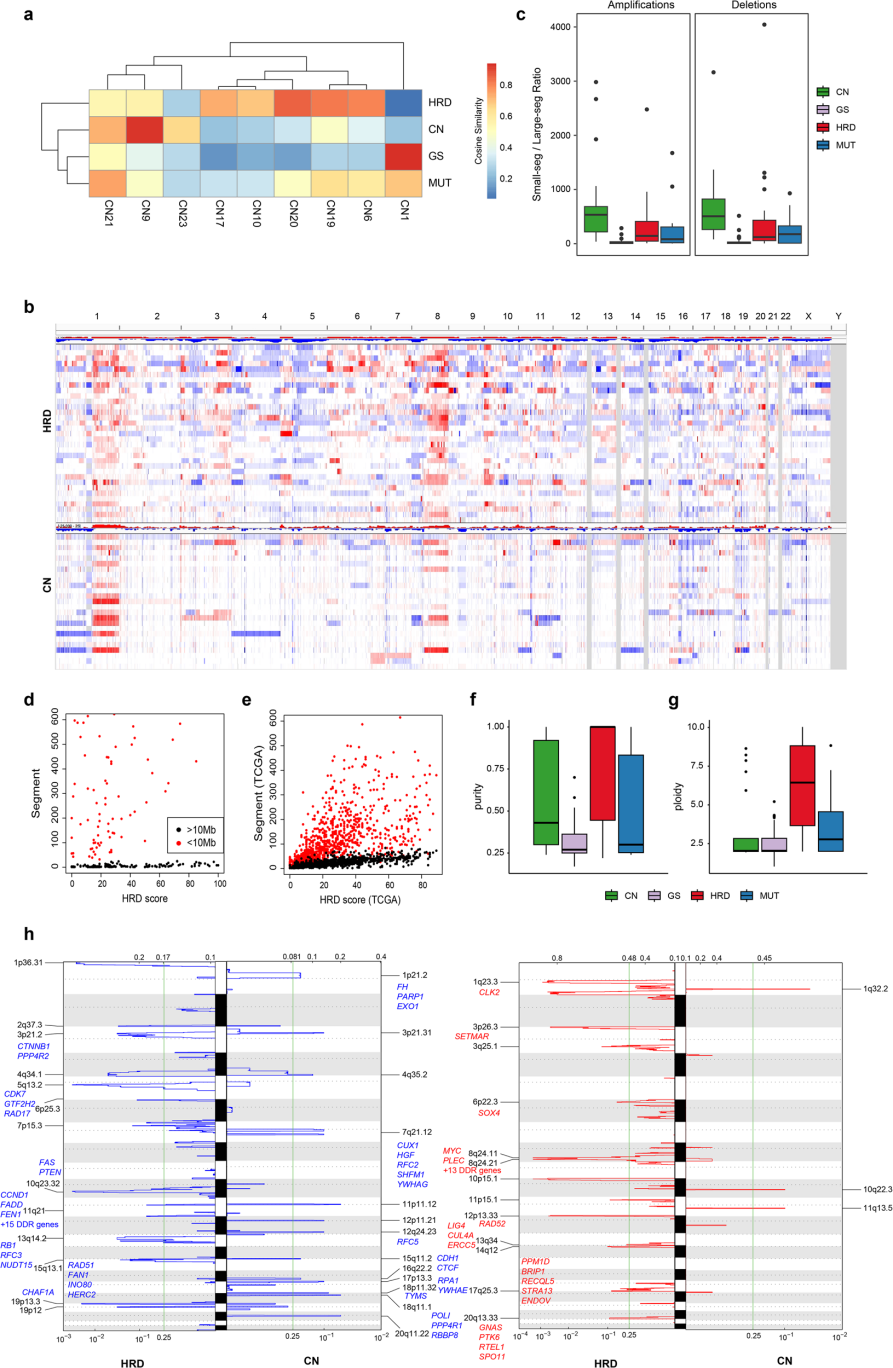
Copy number (CN) alteration landscape and CN signature profiles. **a** Cosine similarity between CN signature profiles and samples across the four subtypes. The CN subtype shows the highest similarity with CN9 (small-sized focal loss of heterozygosity), whereas CN17, CN10, CN20, CN19, and CN5 are more enriched in the homologous recombination deficient (HRD) subtype, reflecting complex genomic instability features. **b** Representative IGV plots showing genome-wide CN patterns in HRD and CN subtypes. Although the two subtypes share a similar overall pattern, the HRD subtype exhibits more visible segments, whereas enrichment in smaller segments within the CN subtype renders alterations less visually prominent. **c** Ratio of small (≤10 Mb) to large (>10 Mb) CN segments is calculated separately for amplifications and deletions across subtypes. The CN subtype exhibits the highest small-to-large segment ratio. **d** Comparison between HRD score and number of CN segments, small segments (red), and distinct large segments (black). **e** The Cancer Genome Atlas (TCGA) cohort confirms the differential distribution of small and large CN segments relative to HRD score, supporting the distinct segmental alteration patterns observed between HRD and CN subtypes in panel **d**. **f** Tumor purity estimates show that the CN subtype tends to have high purity. **g** Tumor ploidy is approximately 2 in the CN subtype, indicating a near-diploid status despite more numerous CN events. **h** GISTIC analysis of focal amplifications and deletions in HRD and CN subtypes. In amplification peaks, there were no genes associated with cancer census genes (CCGs) or DNA damage repair (DDR) in the CN subtype. By contrast, deletion peaks in the CN subtype contained several CCGs. The HRD subtype showed more numerous of DDR genes than CCGs in both amplification and deletion regions. GS genome stable, MUT mutation-dominant.

We examined the abundance of CN mutation signatures in the COSMIC database relative to the four hBC subtypes. In CN subtypes, the CN9 signatures characterized by small-sized (<10 Mb) LOH and heterozygous segments were highly elevated (Fig. 4a). The genome-wide CN profiles show that HRD subtype hBC genomes are prevalent for CN alterations (Fig. 4b). However, the CN subtype of the hBC genomes exhibits more prevalent small-sized CN alterations, including both amplifications and deletions (Fig. 4c). Figure 4d further demonstrates that the burdens of small-sized and large-sized CNs (<10 Mb and >10 Mb, red and black, respectively) show a distinct relationship with HRD scores. Specifically, large CN segments exhibited a gradual increase in abundance with increasing HRD scores, whereas small CN segments were frequently observed even in genomes with low HRD scores. We further explored the genomic distribution of CN profiles relative to the sizes of CNs using public resources of sporadic breast cancers (*n* = 1067, The Cancer Genome Atlas; TCGA) (Fig. 4e). Consistently, large CN segments strongly correlated with HRD scores, whereas small CN segments plateaued at moderate HRD scores, highlighting differential dynamics between small-scale and large-scale CN alterations in breast cancers.

We next assessed tumor purity and ploidy across subtypes (Fig. 4f,g). The genome of the HRD subtype exhibited relatively higher ploidy levels, indicative of widespread chromosomal instability, whereas the ploidy values of the CN subtype were maintained close to diploidy. These findings support the hypothesis that CN and HRD subtype hBC genomes differ not only in the size distribution of CN events but also in the resulting genomic instability, that is, localized versus widespread genomic instability. We conducted GISTIC analyses to identify whether recurrent CNs in CN and HRD subtype genomes involve different cancer-related genes (Fig. 4h and Supplementary Fig. 4). We observed that recurrent deletions in the HRD subtype frequently involve genes representing DDR such as *RAD52*, *MRE11A*, and *STRA13*. Conversely, recurrent deletions in the CN subtype genomes commonly involve known tumor suppressor genes such as *CUX1*, *CDH1*, and *CTCF*. Thus, alterations preferred in the HRD subtype hBC genomes frequently target genome maintenance pathways, whereas CN subtype genomes favor the loss of tumor suppressors. A detailed list of genes corresponding to the identified chromosome arm-level peaks is provided in Supplementary Table 10.

### Frequency of chromothripsis and TP53 loss in HRD subtypes

**Fig. 5:**
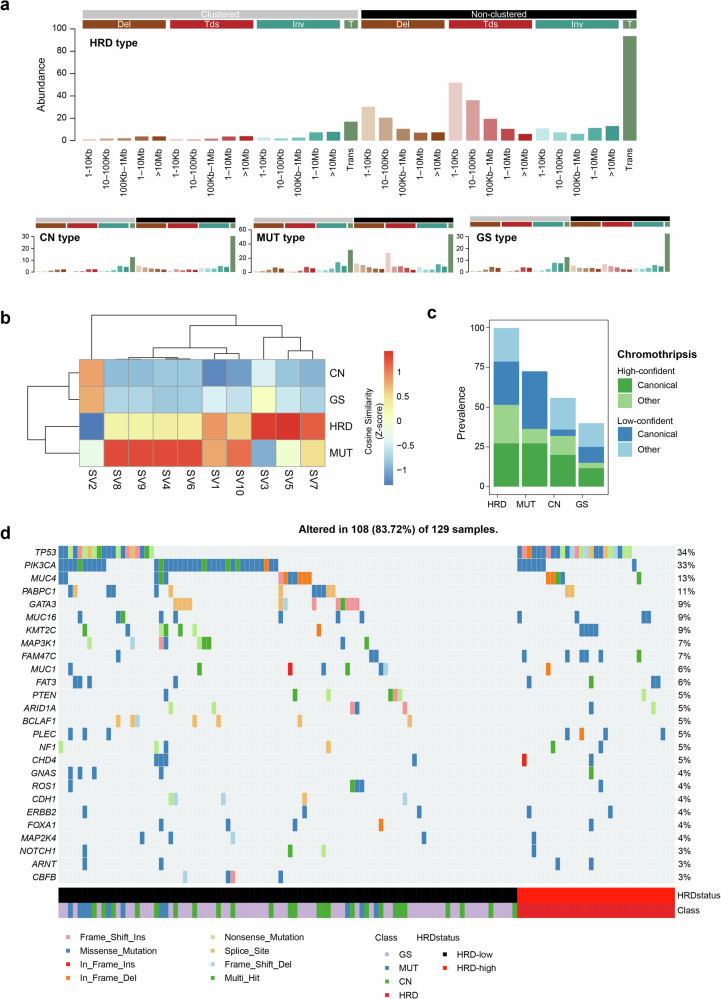
Structural variation and mutation landscape associated with genomic instability in the HRD subtype. **a** Structural variant (SV) signature analysis across subtypes. Although SV signature profiles are generally similar across all subtypes and dominated by SV2, the abundance of this signature is highest in the homologous recombination deficient (HRD) subtype. **b** *Z*-score-normalized cosine similarity of SV signatures reveals higher contributions of SV3 and SV5 in the HRD subtype compared with other groups. **c** Prevalence of chromothripsis events across subtypes. All samples in the HRD group exhibit chromothripsis, whereas 73%, 56%, and 40% of samples in the mutation-dominant (MUT), copy number (CN), and genome stable (GS) subtypes exhibit chromothripsis, respectively. **d** Somatic mutation landscape across the 129-sample cohort. A total of 108 samples harbors at least one somatic variant. TP53 mutations are markedly more frequent in HRD-high samples than in HRD-low ones, whereas PIK3CA mutations are more common in the HRD-low group.

We next examined SV across all four hBC subtypes. Non-clustered translocations were observed as a prominent SV class prevalent across the four types of hBC genomes (Fig. 5a). Non-clustered translocations, the major component of SV2 signatures, are widespread across hBC subtypes except the HRD subtype (Fig. 5a). The HRD subtype hBC genomes specifically harbored SV3, SV5, and SV7 signatures, with SV5 and SV7 featured by non-clustered deletions and observed pan-cancer including breast cancer, and SV3 specifically associated with HRD in breast cancer^53^ (Fig. 5b). The enrichment of SV3 is attributed to relatively short (<100 kb) non-clustered translocations that are specific features of HRD subtype hBC genomes. By contrast, the MUT subtype showed selective exposure to complex SV signatures — SV4, SV6, SV8, and SV9 — with the high cosine similarities (Fig. 5b): SV4 comprises clustered translocations; SV6 and SV9 represent large, mixed rearrangements that frequently co-occur and SV8 corresponds to very short inversions. We further explored whether the HRD subtype is associated with more complex SVs, such as chromothripsis. Chromothripsis is a process involving extensive chromosomal rearrangements occurring in a single catastrophic event^54^. We observed that the frequency of chromothripsis was highest for HRD subtype hBC, and chromothripsis calls were observed in all cases of HRD subtype hBC (Fig. 5c). When somatic mutations were examined between HRD and non-HRD subtype hBC genomes, we found *TP53* significantly enriched in HRD subtype genomes (72.7% (24 of 33) versus 20.8% (20 of 96), *P* = 5.27×10^−7^; Fig. 5d). This suggests that HRD tumors are supplemented with *TP53* losses that might be responsible for the extensive genomic instability of the hBC type^55^.

### Germline variants and transcriptional features in hBC subtypes

**Fig. 6:**
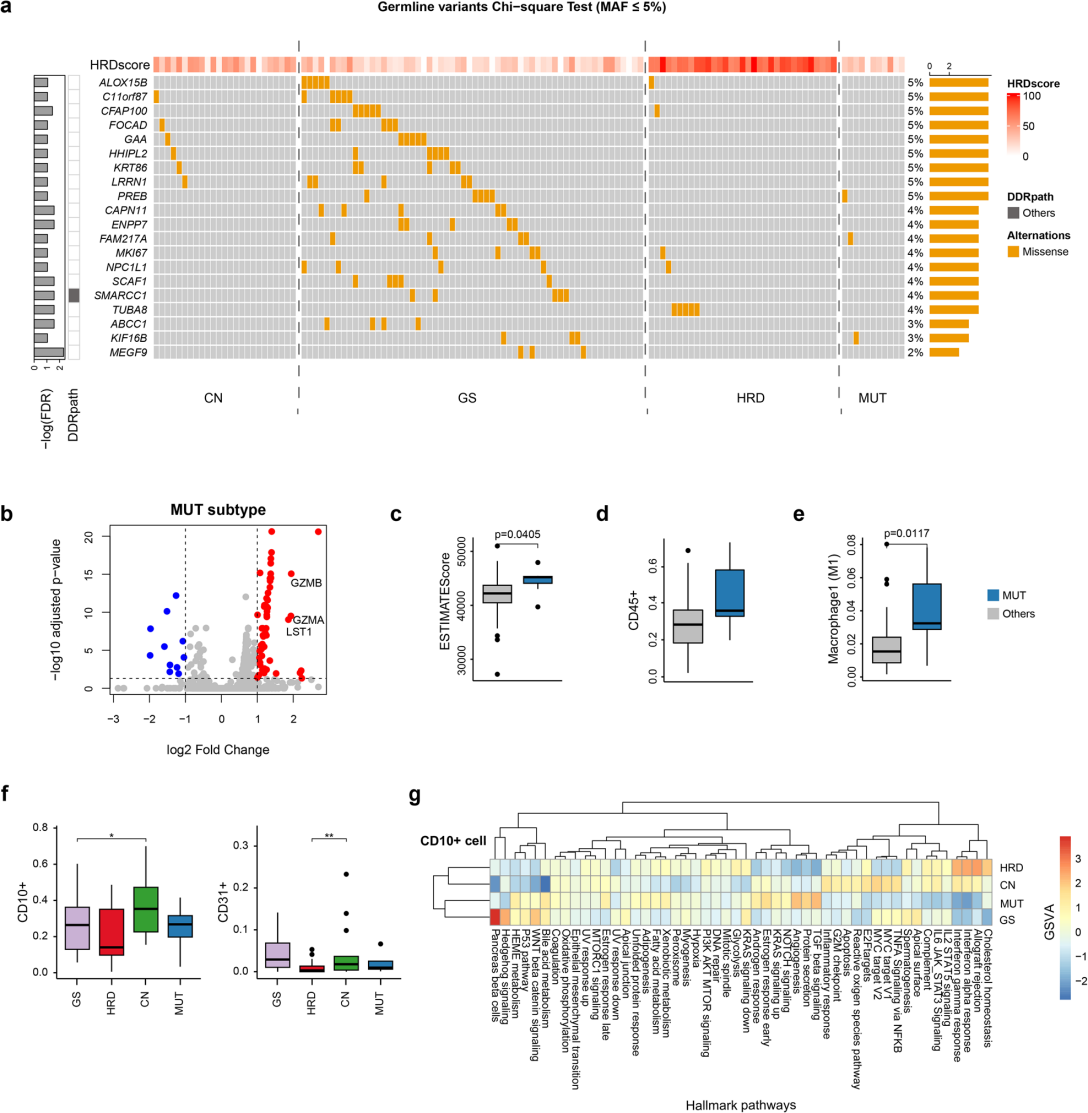
Germline variants and transcriptional features across molecular subtypes. **a** Landscape of germline variants with minor allele frequency (MAF) <5% identified by χ^2^ test (false discovery rate < 0.05) across the four subtypes. The GS subtype exhibits the highest overall germline variant frequency. **b** Gene expression analysis comparing the mutation-dominant (MUT) subtype with all other subtypes revealed a set of genes significantly upregulated in the MUT group, including *GZMB*, *GZMA*, and *LST1*, which are associated with immune activation and cytotoxic functions. Immune microenvironment features of the MUT subtype compared with others. **c** ESTIMATE immune scores. **d** Proportion of CD45⁺ cells. **e** M1 macrophages. **f** Cell-type deconvolution (TR4) analysis reveals significantly larger proportions of CD10⁺ and CD31⁺ cells in the copy number (CN) subtype. **g** Gene set variation analysis (GSVA) from group-wise expression profiles (GEP) of CD10⁺ cells in CN subtypes. The CN subtype showed marked enrichment of proliferation-associated pathways, including MYC targets and E2F signaling. DDR, DNA damage repair, GS genome stable, HRD homologous recombination deficient. TNFA Tumor Necrosis Factor alpha, NFkB Nuclear Factor kappa-light-chain-enhancer of activated B cells.

We examined the hBC subtype-specific germline variants by examining the subtype-specific enrichment of filtered germline calls in hBC genomes (Fig. 6a). Of note, all the significant germline calls (*P* < 0.05; Χ^2^ test) were observed in the GS subtype of hBC genomes, which are relatively deficient in other types of somatic alterations. We observed that *SMARCC1* was identified as a significantly enriched germline variant in GS subtype genomes. According to the ACMG/AMP guidelines, 145 significant germline variants were classified: 1 likely pathogenic, 112 VUS, 25 likely benign, and 7 benign^56^. The single variants classified as likely pathogenic (GAA P361Q) has low cancer relevance and no reported in association with breast cancer. In addition, other DDR-related genes, including *BARD1* (*n* = 7), *ASCC3* (*n* = 7), and *BLM* (*n* = 8), were observed as recurrent germline variants without subtype specificity (Supplementary Fig. 5).

Transcriptome-based gene set enrichment analysis revealed hBC subtype-specific molecular pathways. For example, CN subtype hBC showed a relative enrichment of fibroblast activation pathways representing a cancer-associated fibroblast (CAF)-like phenotype (Supplementary Fig. 6). A strong enrichment of cell cycle and cancer-related pathways was found in the HRD subtype, representing their high proliferative nature. Conversely, cancer-related features were relatively downregulated in GS and MUT subtypes of hBC. We further observed significant upregulation of *GZMA*, *GZMB*, and *LST1*, specifically in the MUT subtype (Fig. 6b and Supplementary Fig. 6), indicating elevated anticancer immune activity. Transcriptome-based profiling of tumor microenvironments further supports a high abundance of immune cells in MUT subtype hBC cases (ESTIMATE scores; Fig. 6c). Deconvolution of four major cellular components (epithelial, fibroblasts, endothelial cells, and immune cells) also revealed that this hBC subtype exhibited the highest levels of infiltrating immune cells (CD45^+^ cells) (Fig. 6d). Further analysis of 22 immune-related features derived from the deconvolution (Supplementary Fig. 7) showed that M1 macrophage levels were significantly elevated in the MUT subtypes (Fig. 6e). Cases of CN subtype hBC exhibited significantly higher abundance of CD10^+^ and CD31^+^ stromal cell populations compared with other hBC subtypes (Fig. 6f). Pathway analysis of subtype-specific gene expression profiles revealed that CD10^+^ fibroblasts in the CN subtype have elevated enrichment of MYC targets, E2F, G2M checkpoints, and inflammatory response pathways, representing elevated stromal activity (Fig. 6g). These findings suggest that CD10^+^ fibroblasts in CN subtype tumors are not only proliferative but also exhibit an inflammatory phenotype, consistent with pro-tumorigenic CAF-like activity within the tumor microenvironment. The pathway analysis results for the other hBC subtype-specific gene expression profiles are provided in Supplementary Fig. 8.

### Prognostic and pharmacogenomic distinctions among hBC subtypes suggest customized therapeutic strategies

**Fig. 7:**
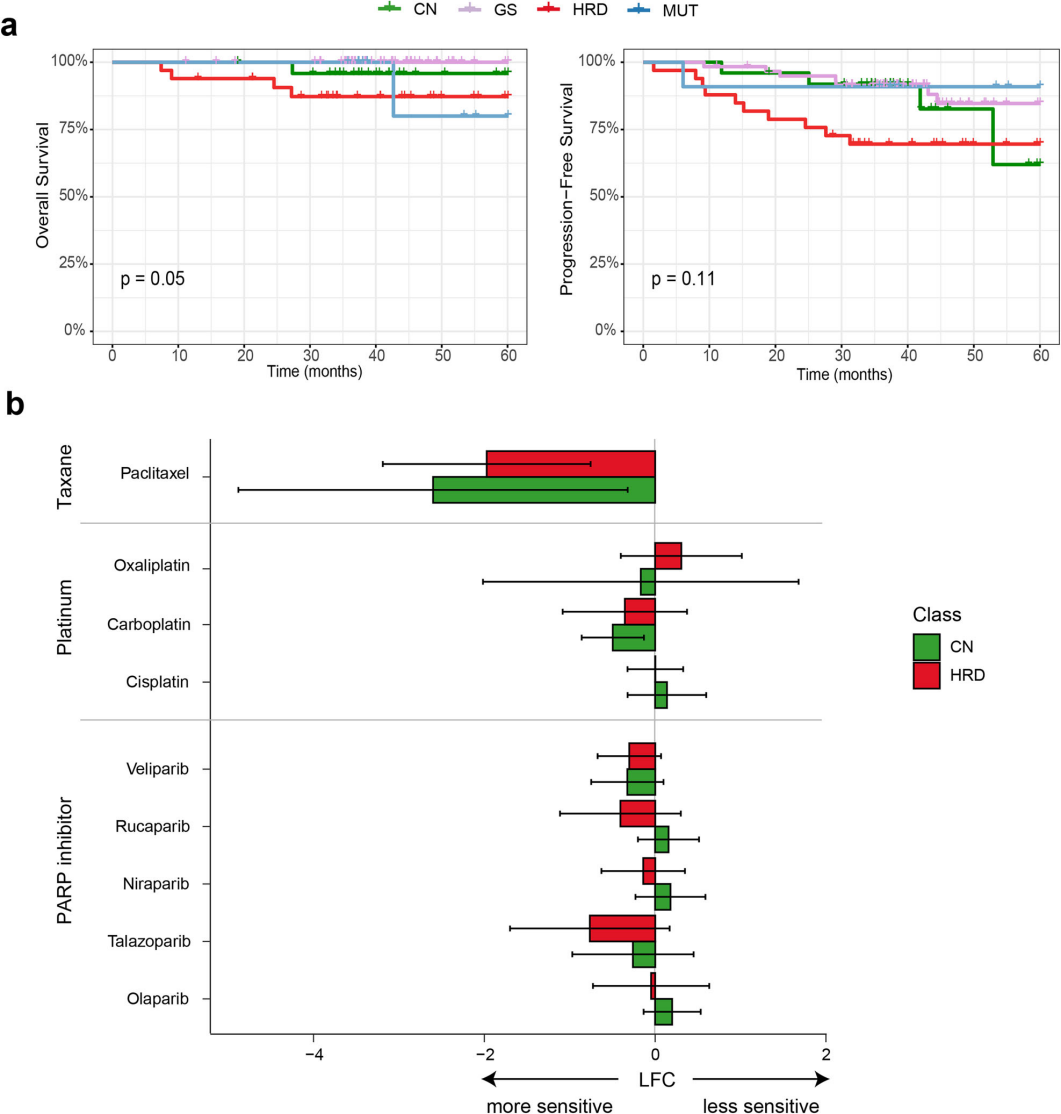
Clinical relevance of molecular subtypes in hereditary breast cancers. **a** Five-year survival analysis of patients by molecular subtype. Kaplan–Meier curves show that the genome stable (GS) subtype is associated with the most favorable outcomes in both overall survival (OS, left) and progression-free survival (PFS, right), whereas the poorest survival is observed in the homologous recombination deficient (HRD) subtype. **b** Drug sensitivity analysis based on molecular subtype. Selected agents include poly (ADP-ribose) polymerase (PARP) inhibitors and chemotherapeutic drugs. A negative log fold change (LFC < 0) indicates higher sensitivity. Most PARP inhibitors show a trend toward greater sensitivity in the HRD subtype. Platinum-based agents tend to be more effective in the copy number (CN) subtype, whereas taxane-based drugs show high sensitivity in both HRD and CN subtypes, with a slightly stronger effect in CN. Extremely low LFC values were considered not biologically significant. MUT, mutation-dominant.

An association between HRD subtype and TNBC has been previously proposed^57^ and is supported by the poor outcomes of the HRD subtype in our 5-year survival analyses (Fig. 7a). The HRD subtype showed an unfavorable prognosis both in overall survival and in progression-free survival. The MUT subtype enriched with luminal B tumors with anticancer immune activity did not show significantly better prognosis compared with the CN and GS subtypes (Supplementary Table 11).

To further investigate therapeutic implications, we used a publicly available cancer cell line data set. A total of 51 breast cancer cell lines was classified into four subtypes by HRD score, as well as by other genomic features, including the burdens of somatic mutations and CN (20 HRD, 13 MUT, 5 CN, and 13 GS subtypes). Then, we examined differential drug sensitivity across the hBC subtypes (Fig. 7b). We focused on a comparison between the HRD and CN subtypes. Although not significant, we observed that the HRD subtype cell lines displayed a trend of increased sensitivity to PARPi, whereas CN subtype cell lines were more susceptible to platinum-based and taxane-based chemotherapy agents (Fig. 7b). These findings are largely consistent with drug sensitivity assays using selected breast cancer cell lines (Supplementary Fig. 9).

## Discussion

In this study, we conducted comprehensive genomic profiling of BRCA1/2-negative hBCs. Despite the absence of these canonical high-penetrance variants, a substantial number of hBC genomes exhibited a high level of HRD. HRD can be detected beyond *BRCA1/2* mutations through integrative analysis of genome-wide mutational signatures, CN alterations, and SV features^58^. Genomic instability was most pronounced in HRD-high tumors, which demonstrated elevated burdens of somatic mutations, CNs, and SVs. However, we also observed that hBC genomes with relatively low HRD scores exhibited considerable genomic instability, particularly in the form of somatic mutations and CN alterations. This observation led us to identify four distinct hBC subtypes, HRD, MUT, CN, and GS, which are representative of different types or absence of genomic instability.

Although prior pan-cancer analyses have suggested that major mechanisms of genomic instability (for example, chromosomal instability and hypermutation) may be mutually exclusive^59^, systematic subtype-level analyses applying this concept to hBC have been limited. Our novel subtype-based approach captures the heterogeneous nature of genomic instability in hBC genomes and demonstrates that multiple, distinct mutational processes can operate or dominate independently of HRD status.

When comparing our genomic subtypes with molecular breast cancer classifications, the HRD subtype was predominantly composed of TNBC, consistent with previous reports linking HRD and *BRCA* losses to this aggressive subset of breast cancers. Of note, it has been largely accepted that HRD-high genomes are susceptible to PARPi owing to the well-established synthetic lethal effect. However, whether conventional chemosensitivity is also assured remains debatable, given the genomic fragility associated with HRD-high genomes^60^. Our study reinforces the clinical relevance of HRD as a predictive biomarker for PARPi, but the response to other chemotherapies, including platinum-based and taxane-based agents, may have differential effects on the HRD-high and CN-prone hBC genomes. Nonetheless, owing to the modest number of cases in each group, validation in larger cohorts will be required.

The identification of mutational signatures revealed that HRD-related signatures predominated in HRD subtype, whereas APOBEC-related signatures were prominent in the MUT subtype, highlighting the differences between the two subtypes. These genomes are also characterized by high TMB correlating with cytolytic immune gene expression, for example, upregulated genes such as *GZMA* and *GZMB* as cytotoxic proteases found in cytotoxic T lymphocytes and natural killer cells and *LST1* involved in immune regulation and inflammatory signaling^61^. In addition, a marked increase in pro-inflammatory M1 macrophages, which are associated with antitumor activity and immune activation and indicate an immunostimulatory tumor microenvironment, was noted in the MUT subtype hBC cases. These findings indicate that the MUT subtype may be amenable to immunotherapy, such as immune checkpoint inhibitors. Previous studies have reported a relative enrichment of M1 macrophage signatures in basal-like subtype^62^, supporting a relationship between immune activation and responsiveness to immunotherapy. Similarly, immunotherapy has demonstrated a clinical response in patients with TNBC. However, recent studies suggest that a subset of HR^+^ patients with breast cancer may also respond to immune checkpoint inhibitors^63–66^. Although it is unclear which specific HR^+^ breast cancer type responds to immunotherapy, our findings indicate the luminal B subtype, predominantly in the MUT subtype, as a promising candidate.

Our study also highlighted two distinct CN configurations between HRD and CN subtypes. In HRD subtypes, *TP53* mutation-driven genomic instability accompanying HRD may contribute to widespread chromosomal instability, resulting in large-scale CN alterations and frequent aneuploidy, consistent with the previous study that *TP53* inactivation tolerates whole-chromosome imbalance and thereby promotes chromosomal instability in human cancers^67^. By contrast, CN subtype hBC genomes are characterized by focal small-sized CN alterations and remain largely euploid, suggesting a unique mechanism of genomic dysregulation. Prior analyses of CN signatures further support that small-segment CN processes and LOH can be uncoupled from high aneuploidy^68^. These tumors harbored deletions in a distinct set of tumor suppressor genes compared with HRD tumors. Moreover, the observed tendency of CN-type cell lines to be more susceptible to platinum-based and taxane-based chemotherapy agents is consistent with previous research indicating that chromosomally unstable cells without accompanying aneuploidy tend to respond better to conventional chemotherapy^69^. However, the activated cancer-associated fibroblast phenotypes observed in CN subtype linked to cytotoxic resistance and adverse outcomes in breast cancer. This suggests that CAF-rich niches may attenuate the chemosensitivity of CN subtype and that stromal-targeted co-interventions (that is, *IL-6/STAT3* axis inhibition) could help restore chemotherapy efficacy in this subtype^70^. These data support that using an integrative metric that combines size-stratified CN status, HRD score, ploidy, and CN-signature exposures as a composite biomarker to guide subtype-specific treatment selection and rational combination strategies in hBC.

The distinct genomic instability patterns observed in our hBC subtypes may, in part, be associated with the presence of extrachromosomal DNA (ecDNA). Both the CN subtype, characterized by focal small-segment amplifications with preserved euploidy, and the MUT subtype, showing prominent APOBEC signatures and frequent kataegis, could reflect potential ecDNA-driven mechanisms. Recent studies have established ecDNA as a major driver of focal oncogene amplification, transcriptional hyperactivation, and intratumoral heterogeneity across cancer types^71^. In glioblastoma, ecDNA-positive tumors exhibit an inverse association with HRD scores; tumors harboring ecDNA maintain relatively low HRD scores despite extensive structural complexity, and those with single-oncogene ecDNA architectures display the lowest HRD levels^72^. This suggests that ecDNA maintenance may be favored by genomically stable backgrounds, potentially paralleling the structural complexity observed in our HRD-low CN subtype. In breast cancer, cyclic ecDNA amplifications at *ERBB2* loci have been identified in ER^+^/HER2^+^ high-risk tumors, emerging early through ER-induced R-loops, APOBEC3B activity, replication stress, and type I interferon signaling^73^, which resembles the focal amplifications observed in our CN subtypes. Moreover, APOBEC3-mediated kataegis has been shown to preferentially localize to ecDNA, rendering it a hypermutable substrate that promotes oncogene diversification and intratumor heterogeneity. This observation is particularly relevant to the elevated frequency of kataegis and the strongest APOBEC signatures observed in our MUT subtypes^74^. Collectively, these findings align with the focal amplification architecture of our CN subtype, the hypermutator phenotype of the MUT subtype, and the HRD-independent genomic complexity across subtypes, suggesting that ecDNA contributes to hBC genomic instability beyond canonical HR and mutation-driven pathways. Considering the potential role of ecDNA in these processes could refine the biological interpretation of our four hBC subtypes and help identify ecDNA-driven vulnerabilities that may be exploited through targeted agents, DNA-damaging therapies, or immunotherapy in selected patient groups.

The GS subtype, while lacking clear markers of genomic instability, was enriched for germline variants in DDR genes, including *SMARCC1*. These may represent low-penetrance susceptibility alleles that contribute to cancer risk in the absence of somatic alterations. Accordingly, these tumors arise primarily through inherited predisposition, requiring fewer somatic events to drive malignancy. Given that most germline variants in GS subtype were classified as VUS under ACMG/AMP, they will be re-evaluated periodically against updated ClinVar database and emerging evidence to ensure accurate clinical interpretation. Importantly, ancestry-related disparities in VUS rates — substantially higher in Asian and Pacific Islander individuals than in Europeans in both hereditary breast and ovarian cancers and Lynch syndrome genes and other cancer genes — underscore that VUS identified in Asian cohorts require ancestry-aware interpretation rather than direct extrapolation from Western (European-ancestry) data sets^75^. From a therapeutic perspective, ~15–20% of patients with breast cancer with HER2 amplification currently qualify for targeted therapies; however, the vast majority remains without molecularly guided options^76^. This gap underscores the importance of exploring additional genomic features that may inform treatment, beyond receptor-defined subgroups. Our findings provide a genomic rationale to broaden precision oncology in hBC. Specifically, PARPi may benefit HRD-enriched tumors, whereas hypermutated (MUT) and CN-driven tumors may respond to immunotherapy or platinum-based chemotherapy, respectively. Notably, immune activation features were observed in the MUT subtype, which is predominantly composed of the luminal B subtype suggesting that this subtype may represent a promising target for immune checkpoint inhibitor therapy. Although the findings should be validated in a larger cohort, these insights move beyond the traditional BRCA-centric paradigm and offer a more refined approach to stratifying patients based on underlying mechanisms of genomic instability.

In conclusion, our study reveals that hBCs without *BRCA1*/*BRCA2* mutations are a molecularly heterogeneous group, exhibiting distinct patterns of genomic alteration that can be leveraged for subtype-specific therapeutic strategies. By integrating germline and somatic alterations, mutation signatures, CN profiles, and immune features, we provide a framework that enables more precise and personalized treatment approaches for patients with hBC.

## Supplementary information

Supplementary Information

Supplementary Tables 1-8 and 10

## Data Availability

Specimens (tissue or blood samples) and data were provided by the NCC Bio Bank of National Cancer Center, Korea. The data used in this study can be found in a public repository: Clinical & Omics Data Archive (CODA) at the National Institute of Health in Korea (accession ID: CODA-R000474; https://coda.nih.go.kr/frt/index.do).
