# Supplementary material for: Delineation of the heterogeneity underlying genomic instability in hereditary breast cancers reveals four disease subtypes
Source: Exp Mol Med. 2026 Apr 16;58(4):1254–68. doi: 10.1038/s12276-026-01693-4 (PMC13144627; doi:10.1038/s12276-026-01693-4)
Supplement: Supplementary file 1 — Supplementary Information [file 12276_2026_1693_MOESM1_ESM.pdf]

Supplementary Fig. 1

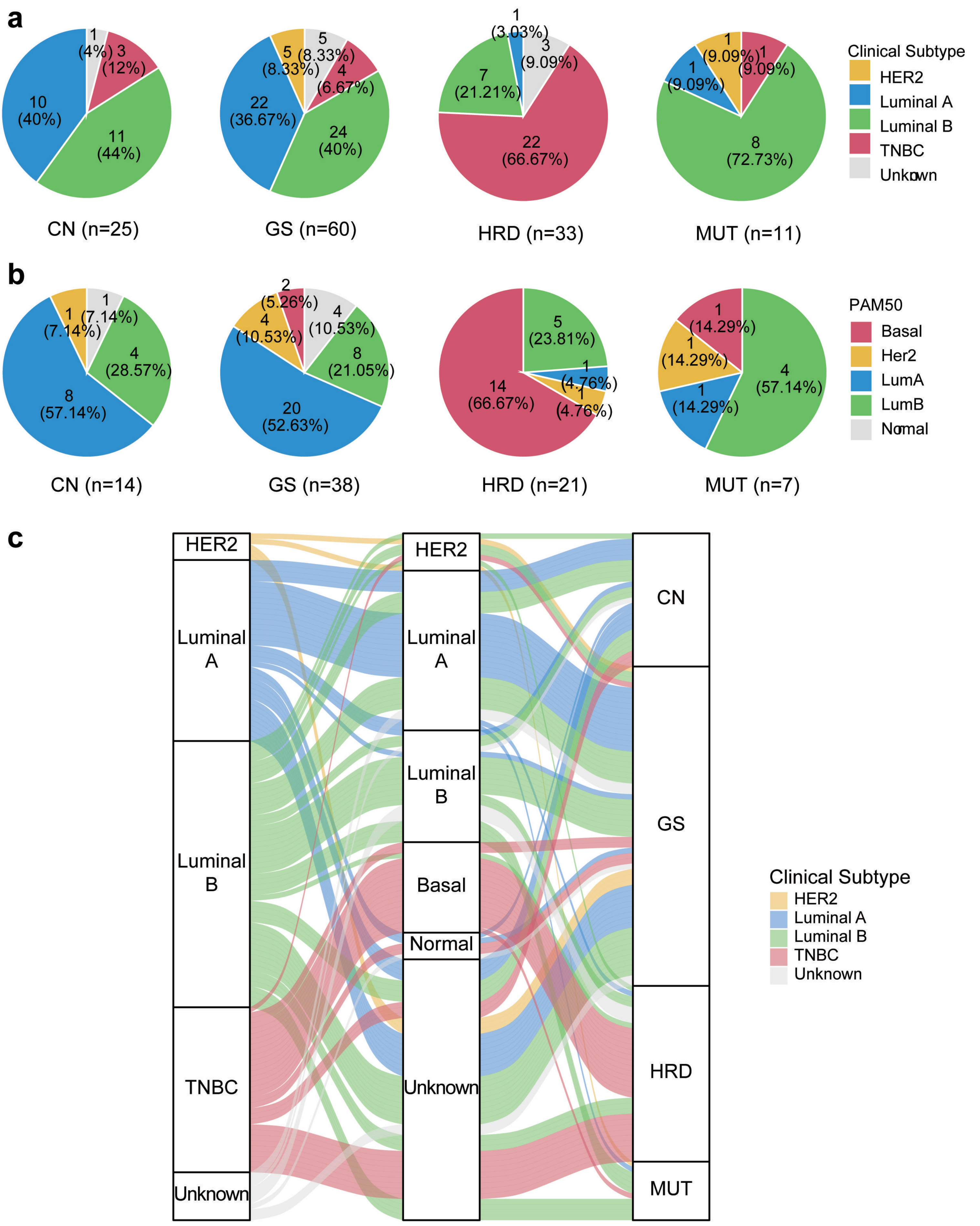

Supplementary Fig. 2

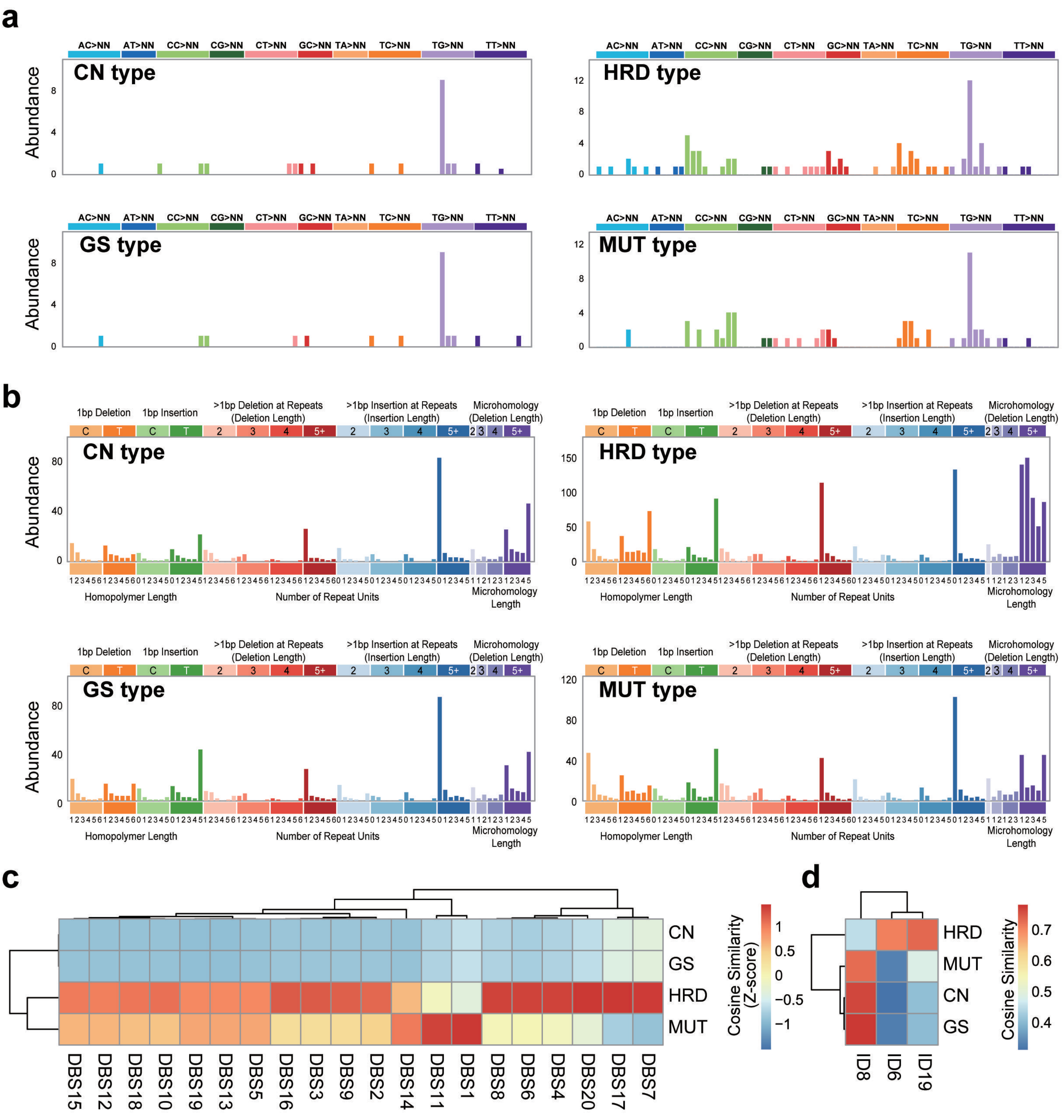

Supplementary Fig. 3

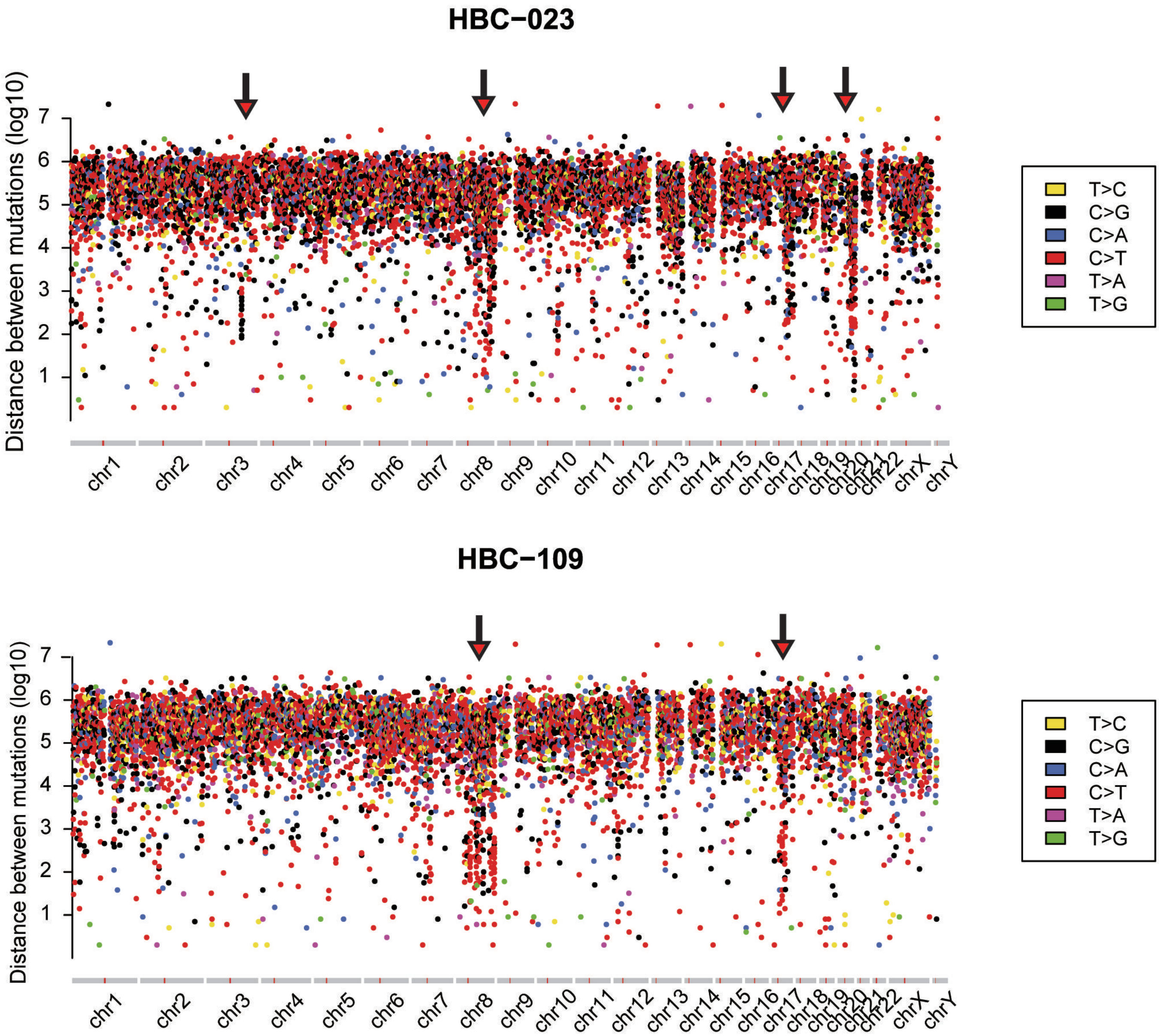

Supplementary Fig. 4

a

HRD

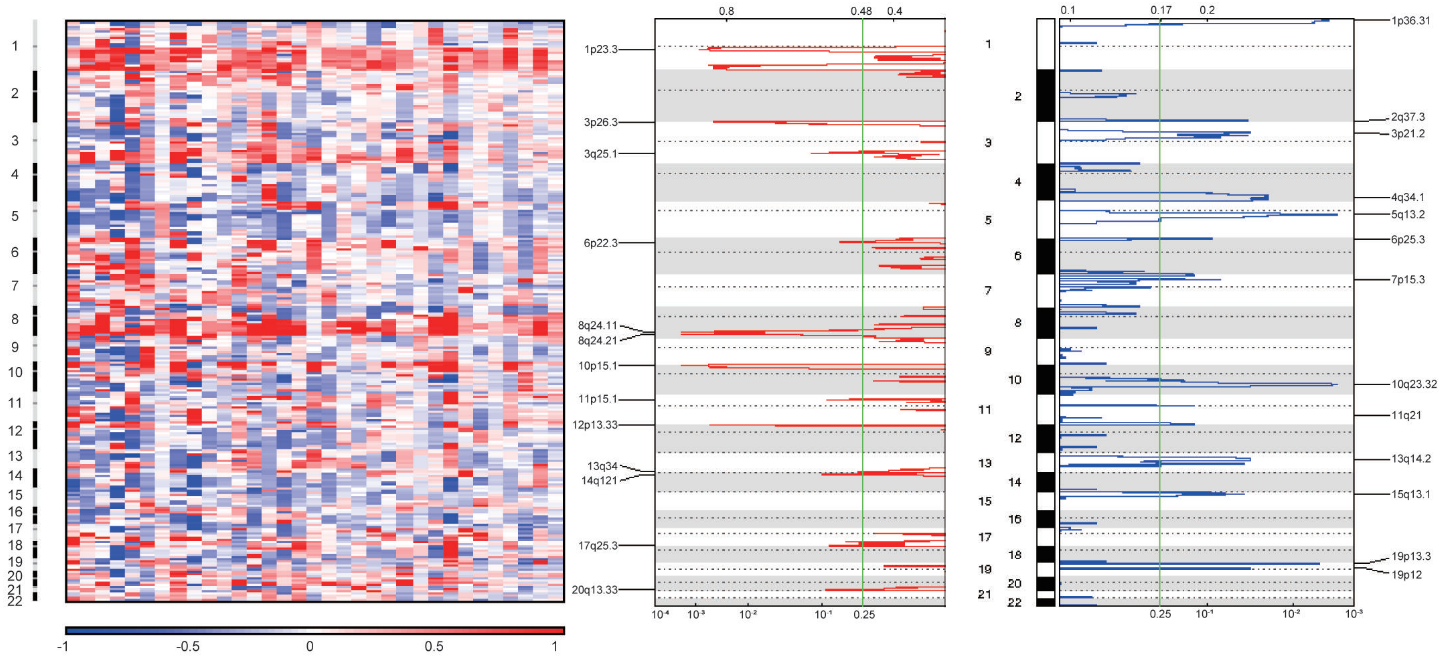

b

CN

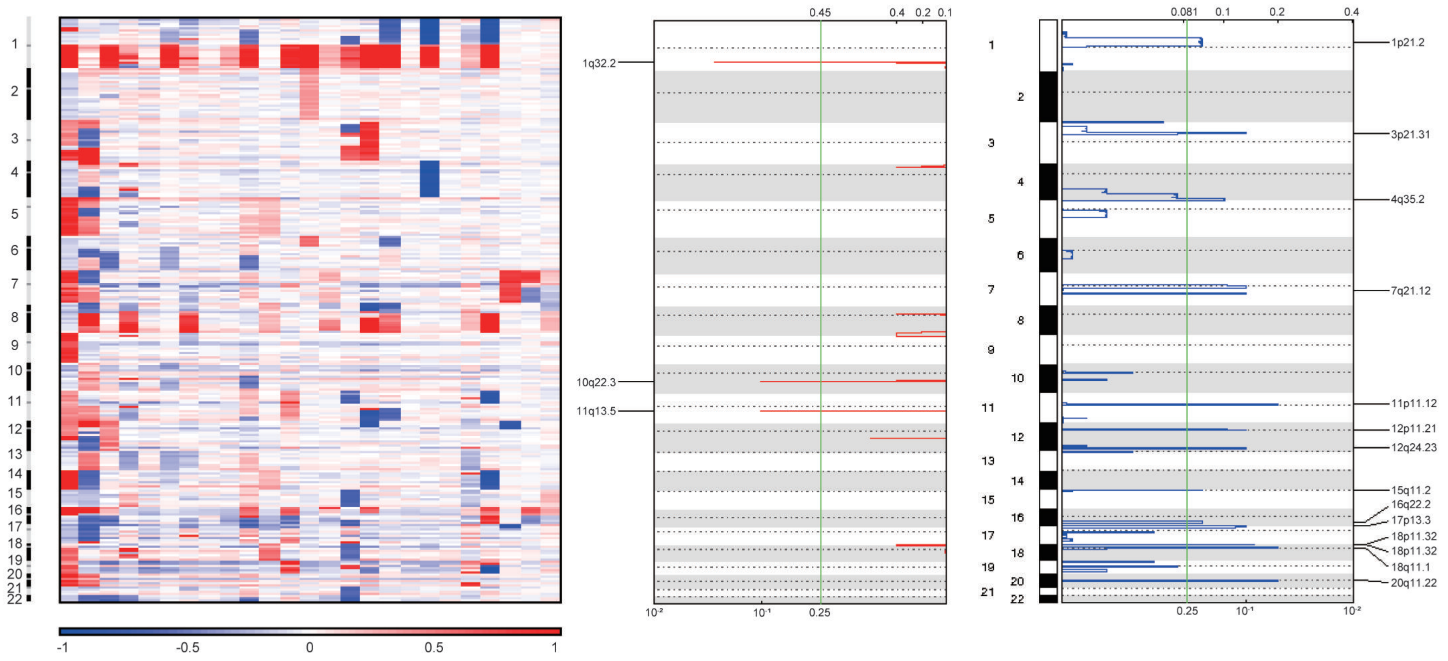

c

MUT

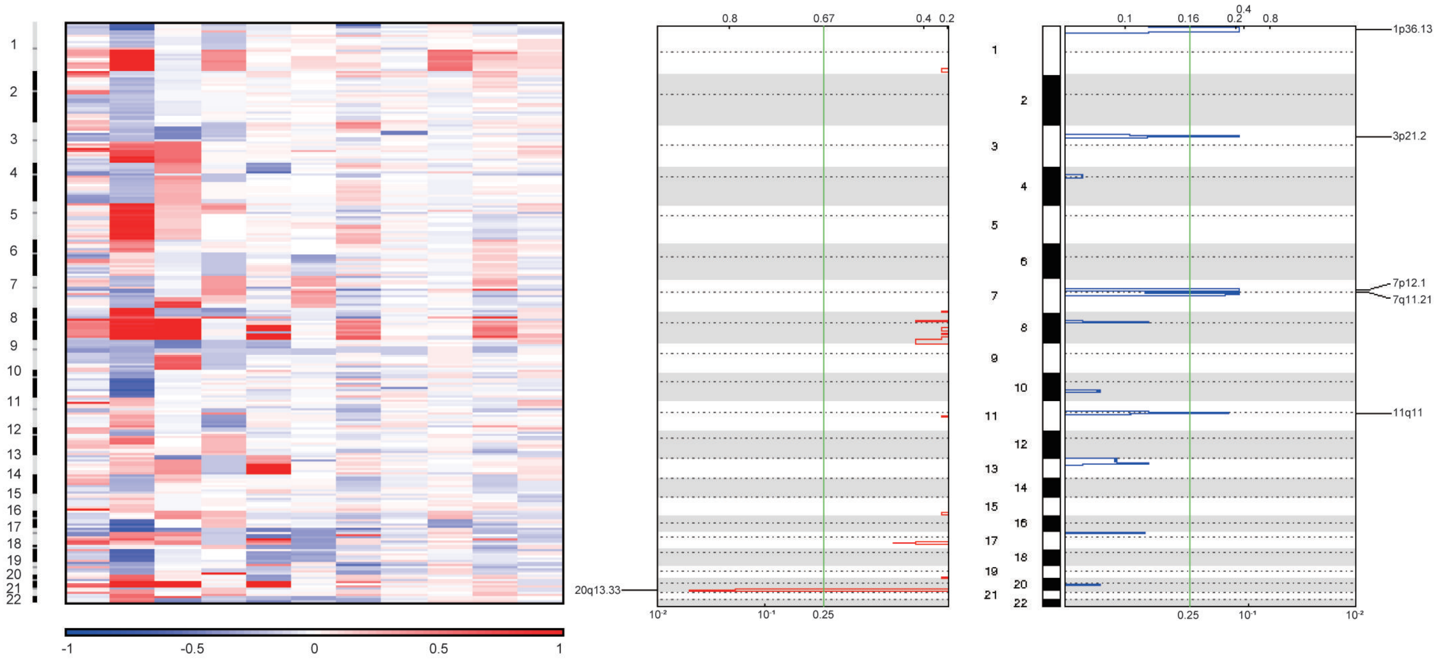

1
2
3
4
5
6
7
8
9
10
11
12
13
14
15
16
17
18
19
20
21
22
-1
-0.5
0
0.5
1
0.8
0.67
0.4
0.2
20q13.33
10⁻²
10⁻¹
0.25
0.1
0.16
0.2
0.4
0.8
1p36.13
3p21.2
7p12.1
7q11.21
11q11
0.25
10⁻¹
10⁻²

d

GS

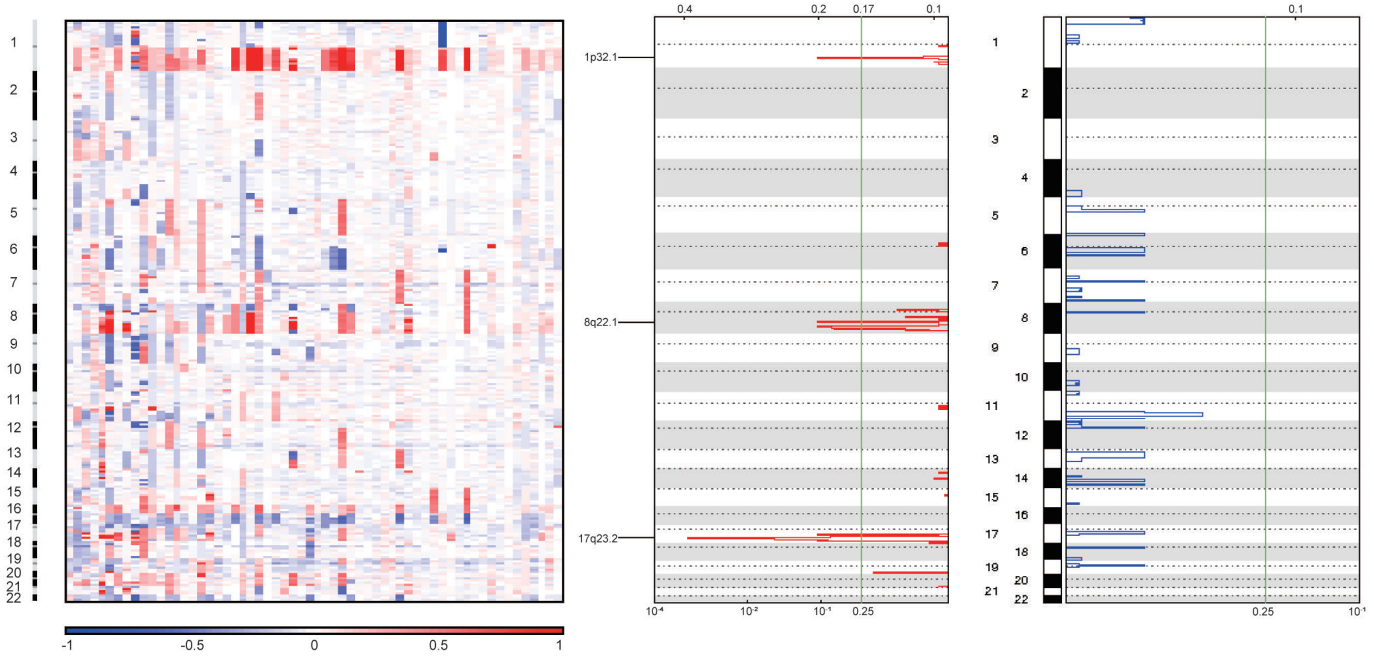

1
2
3
4
5
6
7
8
9
10
11
12
13
14
15
16
17
18
19
20
21
22
-1
-0.5
0
0.5
1
0.4
0.2
0.17
0.1
1p32.1
8q22.1
17q23.2
10⁻⁴
10⁻²
10⁻¹
0.25
0.1
0.25
10⁻¹

Supplementary Fig. 5

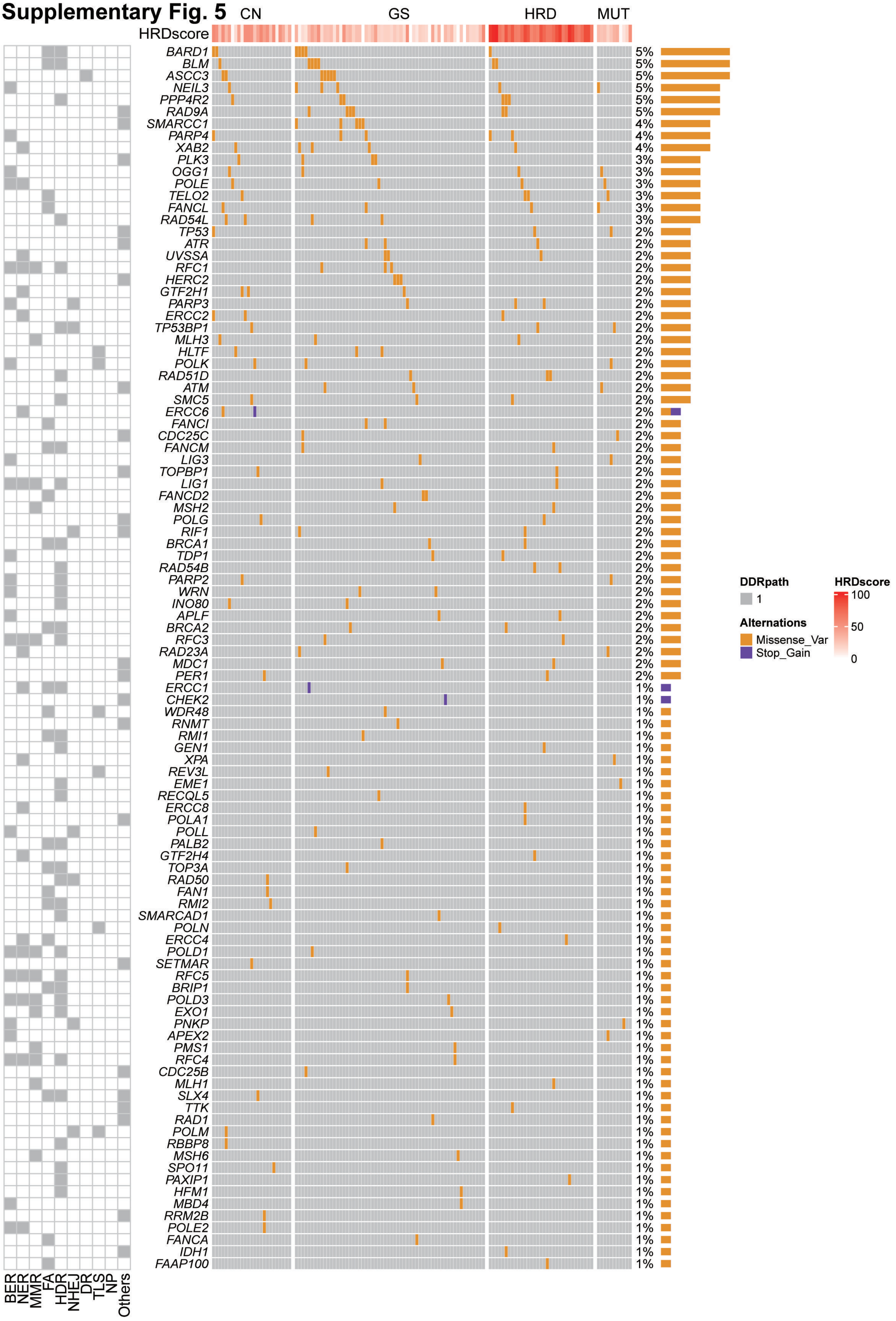

Supplementary Fig. 6

a

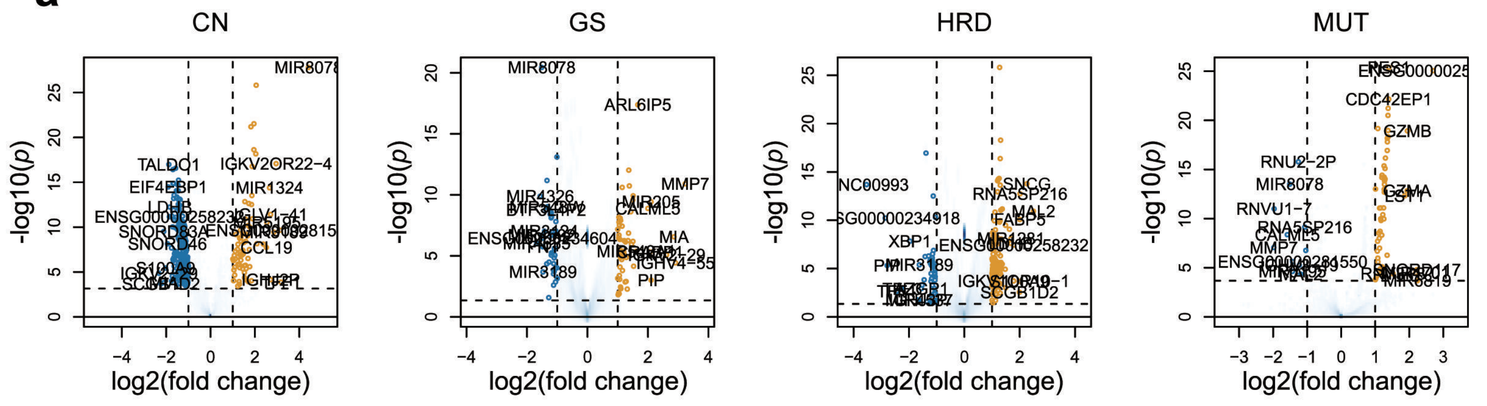

b

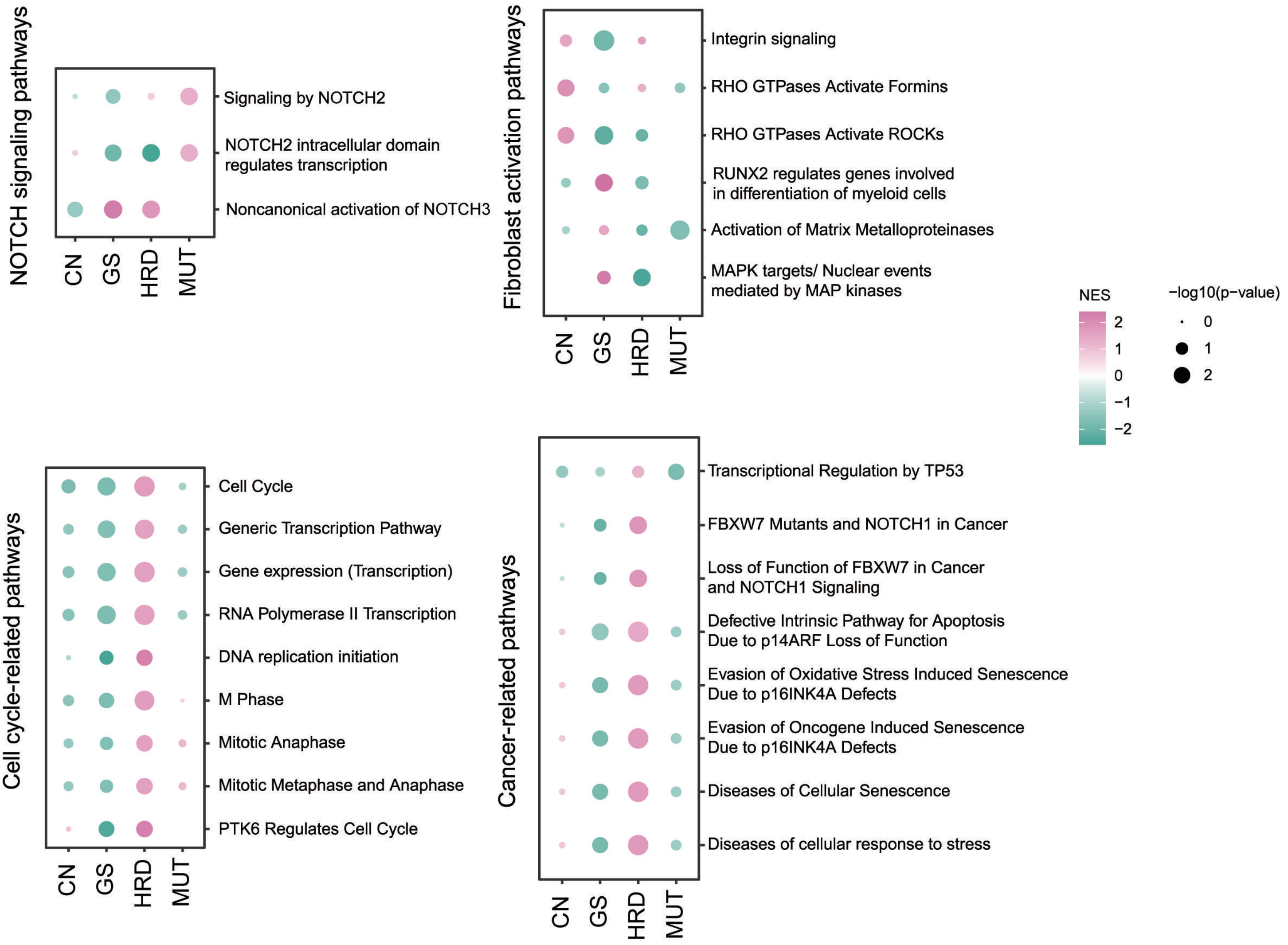

Supplementary Fig. 7

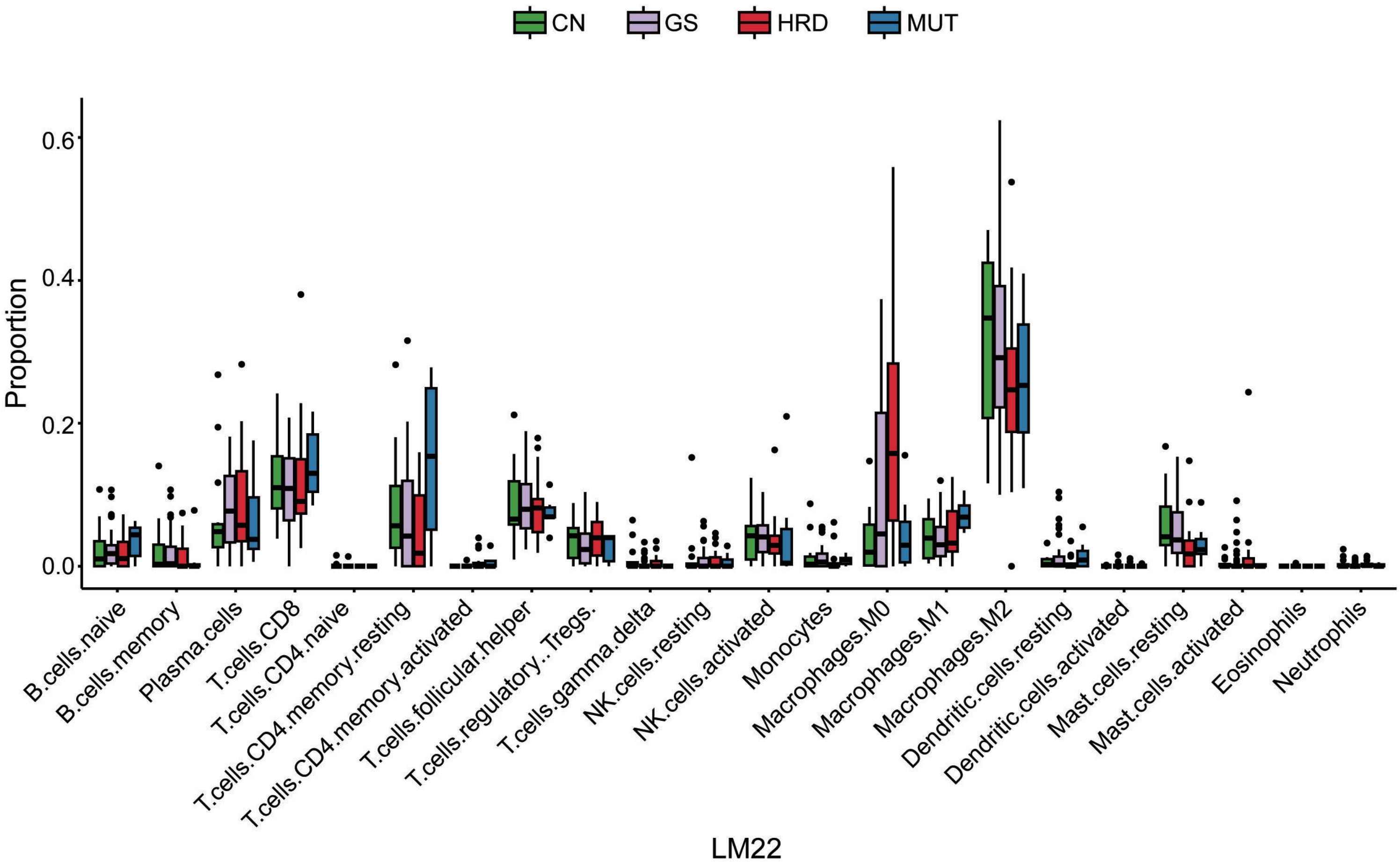

Supplementary Fig. 8

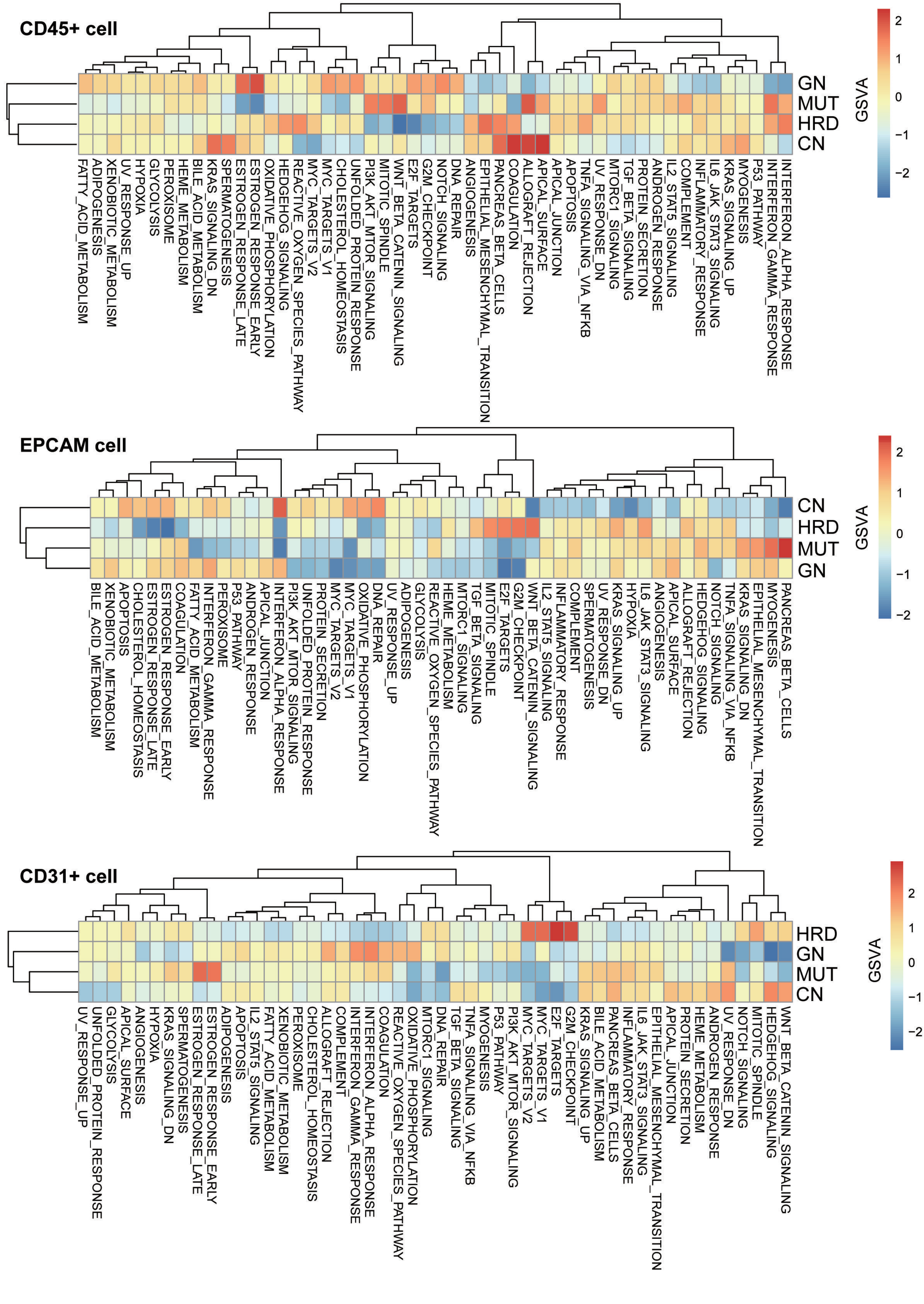

Supplementary Fig. 9

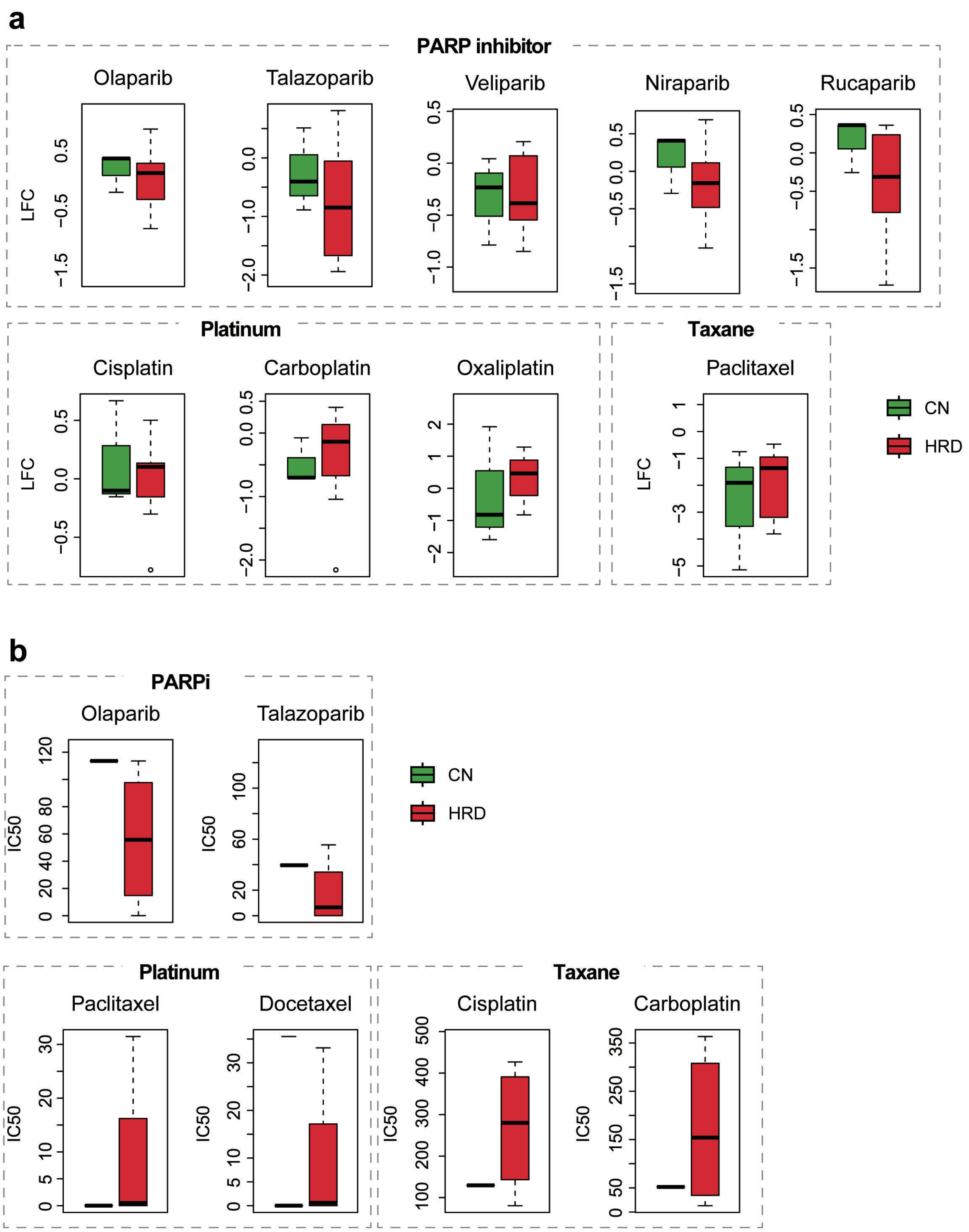

SUPPLEMENTARY INFORMATION

Supplementary Fig 1–9 and Supplementary Table 9 and 11

Supplementary Fig 1. Comparison of hBC subtypes with clinical and PAM50 classifications, related to Figure 2

a-b Distribution of clinical molecular subtype and PAM50 within each four subtypes. **c** Comparison between molecular subtypes and PAM50 classifications reveals that only HRD subtype consistently overlaps with triple-negative breast cancer (TNBC) and the basal-like subtype. Other subtypes show heterogeneous distributions.

Supplementary Fig 2. Double-base substitution (DBS) and indel (ID) signature profiles across hBC subtypes, related to Figure 2.

a-b DBS ID signature profiles across the four subtypes. Bar plots show the relative contribution of individual signature components.

c-d Cosine similarity scores of DBS and ID signatures are presented for each subtype. In ID signatures, only signatures with cosine similarity >0.6 in at least one subtype are shown.

Supplementary Fig 3. Representative examples of APOBEC-associated kataegis in MUT hBC genomes, related to Figure 3.

Examples of APOBEC-associated kataegis in representative MUT subtype. Clustered mutations (arrows) are enriched in the TpCpW trinucleotide context (black and red), indicative of APOBEC activity. The y-axis shows intermutation distance on a log10 scale and x-axis shows genomic position by chromosome.

Supplementary Fig 4. Copy number alteration landscape across the four sugtypes, related to Figure 4.

Genome-wide CNV profiles across all samples in each subtype (**a**: HRD, **b**: CN, **c**: MUT, **d**: GS) are shown. Red indicates amplifications, and blue indicates deletions. Peak regions identified by GISTIC represent statistically significant, recurrent copy number alterations, indicating potential driver genes within amplified or deleted regions.

Supplementary Fig 5. Germline variant profiles of DDR pathway–related genes, related to Figure 6.

Distribution of germline variants in DNA damage repair (DDR)–related genes is shown across the four subtypes. A DDRpath value of 1 indicates that the corresponding gene is included in the DDR pathway.

Supplementary Fig 6. Transcriptome analysis and Reactome pathway enrichment across hBC subtypes, related to Figure 6.

a Differentially expressed genes (DEGs) across the four subtypes. Blue indicates downregulated genes in the specific subtype compared with the other subtypes, while yellow indicates upregulated genes.

b Reactome pathway enrichment analysis of these DEGs, showing that the MUT subtype is enriched in immune pathways associated with NOTCH2 signaling, the CN subtype in stromal pathways related to cancer-associated fibroblasts, and the HRD subtype in cell cycle and cancer-related pathways.

Supplementary Fig 7. Immune cell composition using the LM22 deconvolution, related to Figure 6.

Estimated immune cell proportions for each subtype reveal that the MUT subtype displays higher estimated abundance of CD4+ memory T cells and M1 macrophages compared with other subtypes.

Supplementary Fig 8. Gene set variation analysis (GSVA) from group-wise expression profiles (GEP) derived via TR4 deconvolution, related to Figure 6.

GSVA was performed on GEP of distinct cell types. ($CD45^+$ immune cells, $EPCAM^+$ epithelial cells, and $CD31^+$ stromal/vascular cells). The MUT subtype was characterized by strong activation of immune-response pathways (e.g., interferon-γ and TNFα signaling) in $CD45^+$ cells, indicating immune activation. The CN subtype showed marked enrichment of epithelial-to-mesenchymal transition (EMT), growth signaling, and stromal-response pathways in $EPCAM^+$ cells, indicating a prominent stromal-reactive phenotype. The HRD subtype exhibited a trend toward enhanced DNA repair and proliferation-associated pathways across multiple cell types. In contrast, the GS subtype showed no distinct pathway enrichments, with GSVA scores remaining near baseline.

Supplementary Fig 9. Subtype-specific drug sensitivity patterns in HRD and CN subtypes, related to Figure 7.

a PRISM-CCLE screening data indicate greater sensitivity of the HRD subtype to PARP inhibitors, whereas CN subtype shows higher sensitivity to platinum-based and taxane agents. **b** Validation using breast cancer cell lines confirms these subtype-specific drug response patterns, supporting the reproducibility of the observed therapeutic vulnerabilities.

Supplementary Table 9. Clinical characteristics of study population

Characteristics	N=129 (%)
**Age at diagnosis**	
mean±SD	48.0 ± 11.4
<40	36 (27.9)
40-49	45 (34.9)
50-59	24 (18.6)
≥60	24 (18.6)
**Pathological stage**	
0	7 (5.4)
I	40 (31.0)
II	59 (45.7)
III	10 (7.8)
IV	13 (10.1)
**Subtype**[a]	
Luminal A	34 (26.4)
Luminal B	50 (38.8)
HER2	5 (3.9)
TNBC	31 (24.0)
Unknown	9 (7.0)
**Pathological classification**	
Invasive ductal carcinoma	118 (91.5)
Ductal carcinoma in situ	8 (6.2)
Invasive lobular carcinoma	3 (2.3)
**Family history of cancer**	
Yes	100 (77.5)
BC	40 (31.0)
OC	1 (1.0)
BC and OC	1 (1.0)
No	29 (22.5)
**Follow-up duration (months)**	
Median (range)	41.9 (7.4-257.3)
**Recurrence**	
Yes	27 (20.9)
No	102 (79.1)
**Death**	
Yes	9 (7.0)
No	120 (93.0)

Abbreviations: HER2, human epidermal growth factor receptor2; TNBC, triple-negative breast cancer; BC, breast cancer; OC, ovarian cancer

[a]Subtypes were classified based on the 2011 St. Gallen Consensus, by immunohistochemistry (IHC), silver in situ hybridization (SISH), and fluorescence in situ hybridization (FISH) results.

Supplementary Table 11. Clinical characteristics of study population by subtype

Characteristics	CN (N=25)	GS (N=60)	HRD (N=33)	MUT (N=11)	p-value[c]
**Age at diagnosis**					
mean±SD	49.1 ± 12.2	47.1 ± 11.0	47.6 ± 11.8	51.5 ± 10.9	0.662
<40	6 (24.0)	17 (28.3)	10 (30.3)	3 (27.3)	
40-49	10 (40.0)	24 (40.0)	9 (27.3)	2 (18.2)	0.884
50-59	4 (16.0)	9 (15.0)	8 (24.2)	3 (27.3)	
≥60	5 (20.0)	10 (16.7)	6 (18.2)	3 (27.3)	
**Pathological stage**					
0	3 (12.0)	4 (6.7)	0 (0.0)	0 (0.0)	
I	6 (24.0)	29 (48.3)	11 (33.3)	4 (36.4)	
II	14 (56.0)	20 (33.3)	21 (63.6)	6 (54.5)	0.084
III	2 (8.0)	7 (11.7)	0 (0.0)	1 (9.1)	
IV	0 (0.0)	0 (0.0)	1 (3.0)	0 (0.0)	
**Subtype[a]**					
Luminal A	10 (40.0)	22 (36.7)	1 (3.0)	1 (9.1)	
Luminal B	11 (44.0)	24 (40.0)	7 (21.2)	8 (72.7)	
HER2	0 (0.0)	4 (6.7)	0 (0.0)	1 (9.1)	<0.001
TNBC	3 (12.0)	5 (8.3)	22 (66.7)	1 (9.1)	
Unknown	1 (4.0)	5 (8.3)	3 (9.1)	0 (0.0)	
**Pathological classification**					
IDC	21 (84.0)	53 (88.3)	33 (100.0)	11 (100.0)	
DCIS	3 (12.0)	5 (8.3)	0 (0.0)	0 (0.0)	0.352
ILC	1 (4.0)	2 (3.3)	0 (0.0)	0 (0.0)	
**Family history of cancer**					
Yes	20 (80.0)	43 (71.7)	26 (78.8)	11 (100.0)	
BC	6 (24.0)	20 (33.3)	8 (24.2)	6 (54.5)	
OC	0 (0.0)	1 (3.3)	0 (0.0)	0 (0.0)	0.213[b]
BC and OC	0 (0.0)	1 (3.3)	0 (0.0)	0 (0.0)	
No	19 (76.0)	40 (66.7)	25 (75.8)	5 (45.5)	
**Follow-up duration (months)**					
Median (range)	38.8 (19.0–116.4)	44.5 (11.0–222.6)	40.6 (7.4–257.3)	38.6 (35.7–69.9)	0.599
**Recurrence**					
Yes	4 (16.0)	10 (16.7)	12 (36.4)	1 (9.1)	0.709
No	21 (84.0)	50 (83.3)	21 (63.6)	10 (90.9)	
**Death**					
Yes	1 (4.0)	1 (1.7)	6 (18.2)	1 (9.1)	0.024
No	24 (96.0)	59 (98.3)	27 (81.8)	10 (90.9)	

Abbreviations: HER2, human epidermal growth factor receptor 2; TNBC, triple-negative breast cancer; IDC, invasive ductal carcinoma; DCIS, ductal carcinoma in situ; ILC, invasive lobular carcinoma; BC, breast cancer; OC, ovarian cancer

[a] Subtypes were classified based on the 2011 St. Gallen Consensus, by immunohistochemistry (IHC), silver in situ hybridization (SISH), and fluorescence in situ hybridization (FISH) results.

[b] p-value was calculated by comparing individuals with and without a family history of cancer.
[c] p-values were calculated using one-way ANOVA for continuous variables and the chi-square test for categorical variables.

Supplementary Table 9. Clinical characteristics of study population, related to Figure 1
Supplementary Table 11. Clinical characteristics of study population by subtype, related to Figure 7

Supplementary Tables 1–8 and 10

Supplementary Table 1. Sequencing information, related to Figure 1.
Supplementary Table 2. Somatic coding variants, related to Figure 1.
Supplementary Table 3. Germline variants, related to Figure 6.
Supplementary Table 4. Copy number alterations, related to Figure 1.
Supplementary Table 5. Structural variants, related to Figure 1.
Supplementary Table 6. Mutational signatures, related to Figure 1.
Supplementary Table 7. Transcriptome (TPM), related to Figure 6.
Supplementary Table 8. HRD score, tumor mutation burden, copy number burden, and assigned genomic subtype for each CCLE breast cancer cell line included in the drug sensitivity analysis
Supplementary Table 10. Genes corresponding to GISTIC peak, related to Figure 4.
